# Ribosomes lacking bS21 gain function to regulate protein synthesis in *Flavobacterium johnsoniae*

**DOI:** 10.1093/nar/gkad047

**Published:** 2023-02-02

**Authors:** Zakkary A McNutt, Bappaditya Roy, Bryan T Gemler, Elan A Shatoff, Kyung-Mee Moon, Leonard J Foster, Ralf Bundschuh, Kurt Fredrick

**Affiliations:** Ohio State Biochemistry Program, The Ohio State University, Columbus, OH 43210, USA; Center for RNA Biology, The Ohio State University, Columbus, OH 43210, USA; Center for RNA Biology, The Ohio State University, Columbus, OH 43210, USA; Department of Microbiology, The Ohio State University, Columbus, OH 43210, USA; Center for RNA Biology, The Ohio State University, Columbus, OH 43210, USA; Interdisciplinary Biophysics Graduate Program, The Ohio State University, Columbus, OH 43210, USA; Center for RNA Biology, The Ohio State University, Columbus, OH 43210, USA; Department of Physics, The Ohio State University, Columbus, OH 43210, USA; Department of Biochemistry and Molecular Biology, Michael Smith Laboratories, University of British Columbia, Vancouver, British Columbia, V3T1Z4, Canada; Department of Biochemistry and Molecular Biology, Michael Smith Laboratories, University of British Columbia, Vancouver, British Columbia, V3T1Z4, Canada; Center for RNA Biology, The Ohio State University, Columbus, OH 43210, USA; Interdisciplinary Biophysics Graduate Program, The Ohio State University, Columbus, OH 43210, USA; Department of Physics, The Ohio State University, Columbus, OH 43210, USA; Department of Chemistry & Biochemistry, The Ohio State University, Columbus, OH 43210, USA; Division of Hematology, Department of Internal Medicine, The Ohio State University, Columbus, OH 43210, USA; Ohio State Biochemistry Program, The Ohio State University, Columbus, OH 43210, USA; Center for RNA Biology, The Ohio State University, Columbus, OH 43210, USA; Department of Microbiology, The Ohio State University, Columbus, OH 43210, USA

## Abstract

Ribosomes of Bacteroidia (formerly Bacteroidetes) fail to recognize Shine-Dalgarno (SD) sequences even though they harbor the anti-SD (ASD) of 16S rRNA. Inhibition of SD-ASD pairing is due to sequestration of the 3’ tail of 16S rRNA in a pocket formed by bS21, bS18, and bS6 on the 30S platform. Interestingly, in many Flavobacteriales, the gene encoding bS21, *rpsU*, contains an extended SD sequence. In this work, we present genetic and biochemical evidence that bS21 synthesis in *Flavobacterium johnsoniae* is autoregulated via a subpopulation of ribosomes that specifically lack bS21. Mutation or depletion of bS21 in the cell increases translation of reporters with strong SD sequences, such as *rpsU’-gfp*, but has no effect on other reporters. Purified ribosomes lacking bS21 (or its C-terminal region) exhibit higher rates of initiation on *rpsU* mRNA and lower rates of initiation on other (SD-less) mRNAs than control ribosomes. The mechanism of autoregulation depends on extensive pairing between mRNA and 16S rRNA, and exceptionally strong SD sequences, with predicted pairing free energies of < –13 kcal/mol, are characteristic of *rpsU* across the Bacteroidota. This work uncovers a clear example of *specialized* ribosomes in bacteria.

## Graphical Abstract

**Graphical Abstract:**
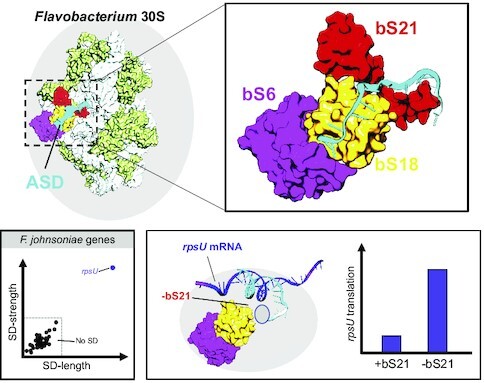
In Flavobacterium, SD-dependent translation is inhibited due to sequestration of the ASD. Yet, translation of rpsU entails an extraordinarily strong SD sequence and is augmented by ribosomes lacking bS21.

## INTRODUCTION

Ribosomes, the machines of protein synthesis, are two-subunit enzymes composed of several large RNA molecules and dozens of distinct proteins. There is growing evidence that ribosome composition can change within a cell or organism (reviewed in (1,2)). Such ribosome heterogeneity can potentially arise in many ways. Ribosomal protein (RP) content can vary due to the absence of a given protein, for example, or its substitution with a paralogous protein. Many organisms have multiple rDNA copies in their genomes which can be divergent in sequence, thus ribosomes may be built from distinct rRNA within the same cell. Ribosomes may also differ in modifications to the RPs or rRNAs. Additionally, damage that ribosomes sustain over time can lead to heterogeneity. Evidence for ribosome heterogeneity has come from multiple research groups and is not generally disputed. However, whether (or in which cases) altered composition results in *specialized* ribosomes, those that preferentially translate certain mRNAs in the cell, remains an open question and a topic of considerable debate (3,4). Recent studies have shown that changes in the concentration of ribosomes (or subunits) will themselves perturb cellular translation in mRNA-dependent ways (5–7). This complicates the interpretation of ribo-seq and proteomic data collected from growing cells, the type of data upon which models of specialized ribosomes are often based.

Some of the best-known examples of ribosome heterogeneity come from bacteria and pertain to zinc homeostasis. A number of RPs including uS14, bS18, bL28, bL31, bL33 and bL36 come in two distinct forms (8,9). One form, termed *C+*, binds Zn^2+^ via signature CXXC motifs; whereas the other form, termed *C-*, does not. The genomes of many bacteria encode both the C+ and C- forms of certain RPs. In *B. subtilis*, C+ forms of uS14, bL31 and bL33 are encoded by *rpsN*, *rpmE*, *rpmGA*, and *rpmGB*; while the C- paralogs are encoded by *yhzA*, *ytiA* and *rpmGC*. The latter three genes are part of the Zur (zinc uptake regulator) regulon and hence are repressed in the presence of zinc (10,11). Ribosomes of *B. subtilis* grown in nutrient rich media contain the Zn^2+^-bound C+ forms of uS14, bL31 and bL33. When cells are starved of zinc, *yhzA*, *ytiA* and *rpmGC* are derepressed, the C- forms of uS14, bL31 and bL33 are produced, and these proteins substitute for their C+ equivalents in the ribosome. This allows Zn^2+^ to be liberated for more critical use and enables new ribosomes to be made with fewer Zn^2+^ ions (12–15). Given the abundance of ribosomes (7000–70 000 per cell, depending on growth rate (16,17)), exchangeable RPs represent a substantial reservoir of Zn^2+^ for the cell. Similar mechanisms of zinc homeostasis have been characterized in *E. coli*, which encodes C+/C- versions of bL31 and bL36 (18–21), and in *Mycobacterium smegmatis*, which encodes C+/C- versions of uS14, bS18, bL28, bL31 and bL33 (22,23). Alternative C- ribosomes are nearly identical to their C+ counterparts with respect to translation activity. Thorough analyses suggest that *M. smegmatis* bS18(C-) ribosomes are modestly compromised in initiation rate (24), and *E. coli* bL31(C-) ribosomes are modestly compromised in elongation processivity and frame maintenance (20). However, there is no indication that these C- ribosomes exhibit distinct mRNA selectivity.

In many bacteria, initiation of translation often involves pairing between a purine-rich Shine-Dalgarno (SD) sequence of mRNA and the pyrimidine-rich anti-SD (ASD) sequence of the 30S subunit. However, in certain lineages, such as the Bacteroidia (formerly Bacteroidetes), SD sequences are not generally employed (25–27). Initiation in these organisms relies on other mRNA determinants, including upstream adenines (positions −3, −6, −12 and −13), reduced secondary structure, and an absence of AUG trinucleotides in the vicinity of the correct start codon (28). Even though they carry an intact ASD at the 3’ end of 16S rRNA, Bacteroidia ribosomes fail to recognize SD sequences *in vivo* and *in vitro* (25,29,30). A cryo-EM structure of the *Flavobacterium johnsoniae* ribosome at 2.8 Å resolution reveals the basis of this ASD inhibition (29). The 3’ tail of 16S rRNA binds a pocket formed by bS21, bS18 and bS6 on the 30S platform, an interaction that precludes rRNA-mRNA pairing. Most contacts to the 3’ tail are formed by amino acids uniquely conserved in the Bacteroidia, implying a conserved mechanism of ASD sequestration across this bacterial class. By contrast, ribosomes of other bacteria lack the platform pocket, and the 3’ tail of 16S rRNA is free to pair with mRNA (29).

Remarkably, in *F. johnsoniae* and many other Flavobacteriales (formerly Flavobacteriia), the gene encoding bS21, *rpsU*, contains a strong SD, unlike virtually all other genes (29). A subset of Flavobacteriales, which includes Chryseobacteria and related genera, have an alternative core ASD (5’-UCUCA-3’), which differs from the canonical ASD (5’-CCUCC-3’) at two positions (underscores). In all these organisms, the fully complementary sequence is seen upstream of *rpsU* (29). This natural covariation provides compelling evidence that translation of at least one gene in Flavobacteriales, *rpsU*, entails SD-ASD interaction. In the other Bacteroidia orders, strong SDs are frequently found upstream of *rpsU* (bS21) and/or *rpsR* (bS18). As mentioned above, bS21 and bS18 both contribute to ASD occlusion on the 30S platform. These observations raise the possibility that SD sequences are used as regulatory elements, enabling bS21 and/or bS18 to control their own translation (29).

**Figure 1.**
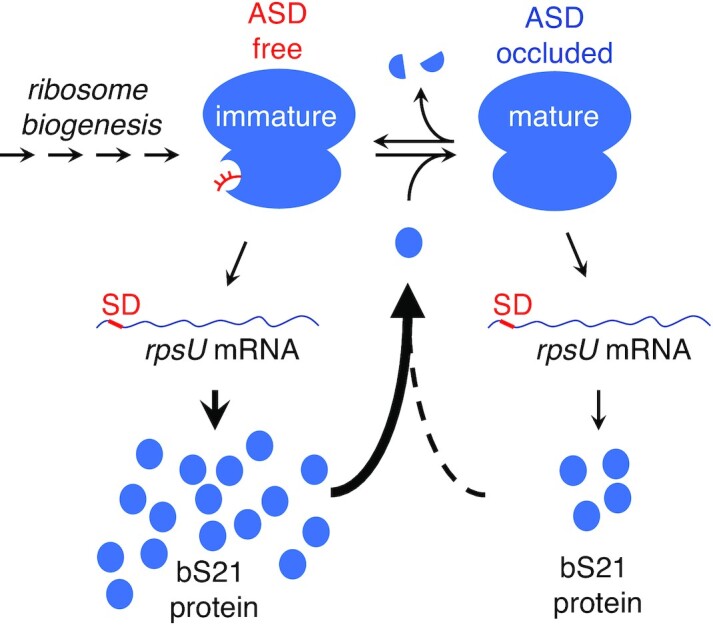
Model of bS21 autoregulation in *F. johnsoniae*. Incorporation of bS21 is one of the last steps in 30S subunit biogenesis. Ribosomes lacking bS21 (immature) contain a liberated ASD, allowing for high-level translation of *rpsU* mRNA. Binding of the product, bS21, generates the replete (mature) ribosome, in which the ASD is occluded. Replete ribosomes will still translate *rpsU* mRNA but at a reduced rate. Loss of bS21 from ribosomes by any mechanism (e.g. targeted proteolysis) would also free the ASD and increase the rate of bS21 synthesis.

Jha *et al.* proposed a simple model of autoregulation of bS21 synthesis in Flavobacteriales (Figure 1). Protein bS21 is one of the last proteins to be incorporated during 30S subunit biogenesis (31–33), and bS21 can dissociate from mature ribosomes (34). Whether formed during assembly or derived from mature ribosomes, Flavobacteriales 30S particles that contain all RPs except bS21 may exhibit translation activity and carry a functionally-liberated ASD. These bS21-lacking subunits may support translation of *rpsU* mRNA at a higher rate than replete subunits, stimulating production of bS21 in the cell. As levels of bS21 rise, so would the proportion of replete ribosomes, consequently damping down the production rate of bS21 (Figure 1) (29).

In this work, we present *in vivo* and *in vitro* evidence that *rpsU* of *F. johnsoniae* is indeed subject to translation autoregulation by ribosomes missing bS21. The mechanism of autoregulation depends on extensive pairing between mRNA and 16S rRNA, and exceptionally strong SD sequences (< –13 kcal/mol) are characteristic of *rpsU* genes across the Bacteroidota phylum, suggesting widespread use of this autoregulatory mechanism.

## MATERIALS AND METHODS

### F. johnsoniae plasmids and strains

Mutations to the *F. johnsoniae* chromosome were made using precise allelic replacement (35). Mutant alleles were generated by separately amplifying ∼1 kb regions from the *F. johnsoniae* chromosome both upstream and downstream of the mutagenesis site. These two fragments were then cloned into the Bam HI and Sph I sites of suicide vector pYT313, using Gibson Assembly (36). Resulting plasmids were moved into *F. johnsoniae*, via tri-parental mating (37), and erythromycin (Em, 100 μg/mL) resistant transconjugants were selected. Colonies were then screened for plasmid integration at the appropriate chromosomal locations using PCR. Confirmed recombinants were then grown overnight in the absence of Em, to allow for loss of the plasmid via a second recombination event, and then cells were plated on 5% (w/v) sucrose, to select against the plasmid. Sucrose-resistant, Em-sensitive colonies were then screened via colony PCR for replacement of the wild-type allele for the mutant allele. Three different *F. johnsoniae* strains were made. In strain ZAM66, the *lacO3-ompA-lacO1* inducible promoter sequence from pZM100 (28) replaces the native *rpsU* promoter. In ZAM64, codons 43–64 of *rpsU* are removed. In strain ZAM65, codon 54 of *rpsU* is changed to GCA (Ala).

The inducible promoter between the Sac II and Spe I sites of pZM100 (28) was replaced by the constitutive *ompA* promoter, using NEBuilder® HiFi DNA Assembly (NEB #E2621S), yielding pZM39. Various TIRs (from positions –25 to +12, where 0 corresponds to the first nucleotide of the start codon) were then cloned into the Bam HI and Xho I sites of pZM39, creating the corresponding *gfp* translational fusion constructs. These constructs were moved into the *F. johnsoniae* strains via tri-parental mating (37). In the case of ZAM66, selection of transconjugants required IPTG (1 mM), because the plasmid encodes LacI. To reduce the promoter distance in pZM46, the plasmid was digested with Spe I and Bam HI, and the ends were bridged by ligation of a short oligonucleotide duplex, yielding plasmid pZM50.

### Growth measurements of *F. johnsoniae*

To measure growth in liquid culture, cells from overnight cultures were diluted 100-fold into casitone yeast extract (CYE) medium (37). When indicated, Em was present at 100 μg/mL and IPTG was present at 1 mM. Cultures were shaken at 250 rpm at 30°C, and aliquots were regularly removed to measure optical density at 600 nm (OD_600_).

To evaluate growth on solid media, overnight cultures were serially diluted, and cells were spotted (20 μL) onto CYE plates, without or with erythromycin (100 μg/mL) and IPTG (1 mM), as specified. Plates were incubated at 30°C for 2 days.

### GFP measurements

Levels of GFP were quantified as described previously (28). Cells from overnight cultures were diluted 100-fold into CYE medium (3 mL) with Em (100 μg/mL), and cultures were grown at 30°C until an OD_600_ of ∼0.5 was reached. For the depletion strains, cells were grown with or without IPTG (1 mM). Cells were pelleted, washed twice with PBS [137 mM NaCl, 2.7 mM KCl, 10 mM Na_2_HPO_4_, 1.8 mM K_2_HPO_4_ (pH 7.4)], and resuspended in the same buffer. The fluorescence of each cell suspension was measured with a Fluorolog-3 (HORIBA) spectrofluorometer, using an excitation wavelength of 481 nm and emission wavelength of 507 nm. Background, determined by measuring fluorescence of untransformed *F. johnsoniae* grown in parallel, was subtracted in each case. Relative Fluorescence Units (RFU) represent fluorescence intensity per OD_600_.

### Purification of ribosomes, subunits, translation factors and bS1.

*F. johnsoniae* strains were grown in CYE media (1 L) at optimum temperature (30°C) to mid-logarithmic phase. Flasks were placed on ice to cool, and cells were pelleted. Ribosomes or 30S subunits were then purified via conventional sedimentation methods as detailed previously (38,39), except that sucrose cushions contained NH_4_Cl at a concentration of 0.3 M rather than 0.5 M and the dialysis step was omitted from the 30S subunit preparation protocol. *F. johnsoniae* initiation factors and *E. coli* EF-Tu were purified as described (29).

The *rpsA* gene was amplified from the *F. johnsoniae* chromosome and cloned into pET28b (Novagen). The resulting plasmid, encoding bS1 with a removable *N*-terminal hexa-histidine tag, was transformed into strain BL21/DE3. Transformed cells were grown in LB media (2.5 L) containing kanamycin (50 μg/mL) at 37°C to mid-log phase, induced with IPTG (1 mM), and grown for an additional 6 h. The overproduced protein was purified from the soluble cell lysate. bS1 was partially purified using Ni-NTA (Qiagen) resin, subjected to thrombin cleavage as described (29), and then further purified using a heparin column (HiTrap, GE Healthcare) and a size exclusion column (Superdex 75, Cytiva). The purified bS1 was dialyzed against storage buffer (50 mM Tris–HCl pH 8, 100 mM NH_4_Cl, 0.1 mM EDTA, 2 mM β-ME, 5% glycerol), distributed into small aliquots, flash frozen, and stored at −80°C.

### Mass spectrometry

Pellets of TCA-precipitated ribosomes or subunits were solubilized in 2% SDS, 100 mM Tris–HCl pH 8 and reduced and alkylated (40) prior to running shortly onto 10% SDS-PAGE resolving gel. The entire lanes were excised and digested with trypsin (Promega), omitting the reduction and alkylation in-gel step (41). Resulting peptides were cleaned on C-18 STop And Go Extraction (STAGE) Tips (42) using 40% acetonitrile and 0.1% formic acid elution buffer and the peptide concentration was estimated using NanoDrop One absorbance at 205 nm, scopes method (ThermoFisher). Either 25ng or 250ng of peptides were analyzed on a quadrupole–time of flight mass spectrometer (Impact II; Bruker Daltonics) coupled to an Easy nano LC 1200 HPLC (ThermoFisher Scientific) using 25 cm × 75 μm 1.6 μm C18 Aurora Series Gen2 analytical column (Ion Opticks, Australia) heated to 50°C by in-house built column heater. Standard 90min peptide was done with the instrument set to acquire the same as (32).

Acquired data was searched on MaxQuant 1.6.17.0 (43) using *F. johnsoniae* protein sequences downloaded from Uniprot (Uniprot.org), NCBI (ncbi.nlm.nih.gov), customized mutant sequences, and the built-in common contaminant sequences (total: 10 368 sequences, downloaded 16 July 2021). The search setting was set with variable modifications of oxidation of methionines and acetylation of protein *N*-termini, and fixed modification of carbamidomethylation of cysteines. Label-free quantitation, match-between-runs, and iBAQ modes were enabled and the protein and peptide false discovery rates were set to 1%. Datafiles and search materials are available via ProteomeXchange with identifier PXD036353 (www.ebi.ac.uk/pride/archive).

For proteins S1–S20, LFQ values were normalized with respect to the median value for each sample. S21 levels were evaluated separately, using two N-terminal peptides (MLIIPIK, MLIIPIKDGENIDR), encoded in all strains and detected in all samples. For each sample, the LFQ values of these two peptides were added. All the resulting sums were then normalized to the WT median sum. Significant differences from WT were evaluated using an unpaired two-tailed *t* test.

### In vitro transcription-translation reactions

PCR products containing *rpsU’-gfp* or *tuf’-gfp* were amplified from plasmids pZM46 and pZM39, respectively, and purified using the GeneJET PCR Purification Kit (Thermo Scientific™ #K0701). A T7 promoter sequence was included in the 5’ portion of each forward primer, and the sequence complementary to the T7 terminator was included in the 5’ portion of each reverse primer. A customized PURExpress® In Vitro Protein Synthesis Kit, lacking ribosomes and initiation factors, was purchased from New England Biolabs (NEB). Transcription-translation reactions were performed according to NEB protocol and supplemented with the following components from either *E. coli* or *F. johnsoniae* (final concentrations indicated): IF1 (2.7 μM), IF2 (0.4 μM), IF3 (1.5 μM) and ribosomes (2 μM). Translation products were labeled by inclusion L-[^35^S]-methionine (Perkin Elmer #NEG009A; 1 μL per reaction). The concentration of DNA template depended on the experiment, as specified in the legend. Translation products were separated by SDS-PAGE (4–20% polyacrylamide gel, BIO-RAD 4561096) and imaged with a Typhoon FLA 9000 phosphorimager (GE Healthcare) and associated software (ImageQuant 5.2).

### tRNA and mRNA

Native *E. coli* tRNA^Lys^ and tRNA^Phe^ were purchased from Chemical Block Ltd. (Moscow, Russia) and charged with lysine and phenylalanine, using purified synthetases, as described (44). Wildtype tRNA^fMet2^ was overexpressed in *E. coli* strain B105, purified using native gel electrophoresis, and charged and formylated as described (44–46).

A DNA template containing a T7 promoter upstream of the *F. johnsoniae* tRNA^Leu^ gene was synthesized using overlapping primers and PCR. This template was used in a 1 mL *in vitro* transcription reaction to generate unmodified tRNA^Leu^, which was then gel purified and charged (29,44).

Model mRNAs were made by *in vitro* transcription and purified by PAGE, as described previously (29,47). To make the plasmid template for the *rpsU* mRNA (mFjoh_1933), a DNA fragment extending from the predicted transcriptional start site (25 nt upstream from the start codon) to codon 30 was amplified and cloned downstream of a T7 promoter in the polylinker region of pUC19. The resulting construct was digested with Bam HI prior to transcription, resulting in a run-off transcript of 137 nt, which includes a primer-binding site (GGUUUUUCUUCUGAAGAUAAAG) at the very 3’ end (29).

### Dipeptide assays

Dipeptide formation was measured using electrophoretic TLC (eTLC) (48). Different reaction schemes were used to deduce rates of decoding or initiation, as outlined in the relevant legend. To measure decoding (scheme 1), the 70S IC was pre-formed by incubating 70S ribosomes (1 μM), mRNA (0.5 μM), formyl-[^35^S]-Met-tRNA^fMet2^ (0.1 μM), IF1 (1.5 μM), IF2 (1.5 μM), IF3 (1.5 μM),and GTP (1 mM) in TNKM buffer (50 mM Tris–HCl pH 7.5, 110 mM NH_4_Cl, 30 mM KCl, 7 mM MgCl_2_, 1 mM DTT) at 37°C for 10 min. Separately, ternary complex (TC) was pre-formed by incubating Leu-tRNA^Leu^ (5 μM), EF-Tu (5 μM), GTP (1 mM), phosphoenolpyruvate (2 mM), and pyruvate kinase (Sigma, 50 μg/mL) in TNKM buffer at 37°C for 5 min. These pre-formed complexes were then re-equilibrated to the appropriate temperature (specified in figure and/or legend) and equal volumes were mixed at time *t* = 0. The reaction was quenched at various time points with 0.5 M KOH. Products were resolved by eTLC and quantified using a Typhoon FLA9000 phosphoimager (GE Healthcare) and associated software (ImageQuant 5.2). To measure initiation rates (schemes 2 and 3), the experimental setup was identical except that one component (mRNA or fMet-tRNA, respectively) was pre-incubated with the TC rather than with the other initiation components. In some experiments (specified in legend), polymix buffer [5 mM potassium phosphate (pH 7.3), 95 mM KCl, 5 mM NH_4_Cl, 5 mM Mg(OAc)_2_, 0.5 mM CaCl_2_, 8 mM putrescine, 1 mM spermidine, 1 mM DTT] was used instead of TNKM buffer.

### Toeprinting assays

Formation of 70S and 30S complexes on various mRNAs was analyzed by toeprinting, as detailed previously (29). Unless otherwise indicated, ribosomes or subunits (1 μM) were incubated with *F. johnsoniae* initiation factors (1.5 μM each, as indicated), mRNA (0.02 μM; with pre-annealed radiolabeled primer), and fMet-tRNA (variable, 0.05–2 μM, as indicated) in TNKM buffer at 37°C for 5 min, and then reactions were subjected to primer extension analysis. The amount of complex formed was quantified (F, fraction of mRNA bound), plotted as a function of fMet-tRNA concentration, and fit to the equation *F* = *F*_max_·[*bc*/(*bc* + 1/*K*_A_)], where *b* is the input tRNA concentration, *c* is the input ribosome or subunit concentration, *K*_A_ is the overall equilibrium association constant, and *F*_max_ is the maximal level of complex detected.

### Selection of genomes

A list of bacterial organisms designated as ‘species representative’ and with an NCBI assembly level ‘complete genome’ was obtained from the Genome Taxonomy Database (GTDB) (49). Genomes and annotations were obtained from NCBI, using the RefSeq assembly summary file available on NCBI’s file transfer protocol system. 4465 such organisms were identified (Table S1).

### Determination of extended ASD sequences

To extract 3’ tail sequences from genomes, we employed a highly conserved 16S rRNA motif (AAGTCGTAACAAGGTAGCCGT) located a fixed distance upstream of the ASD in all examined organisms. First, rRNA sequences were predicted from each genome using barrnap v0.9 (T. Seemann, 2018) with default settings. For each 16S rRNA barrnap prediction, the sequence region from 100 nt upstream of the 3’ end to 10 nt downstream of the 3’ end was extracted. CutAdapt v3.4 (50) was run on the extracted sequence with the settings [-g AAGTCGTAACAAGGTAGCCGT -e 0.25 -O 21 –discard-untrimmed] and the sequence positioned 19–32 nucleotides downstream was isolated as the extended ASD (i.e. 16S nucleotides 1532–1544). To ensure high quality data, ASDs from every 16S rRNA annotation were collected and organisms with more than one unique ASD sequence were removed from the analysis. A single unique ASD sequence was identified in 4364 organisms (Table S1).

### Identification of ribosomal protein genes

In the 4364 organisms with an unambiguous ASD sequence, ribosomal protein genes were selected to evaluate mRNA–rRNA pairing free energies. Genes were identified from the organism's GFF annotation file using common S and L protein metadata annotations. Due to annotation inconsistencies in handling the identical proteins bL7 and bL12, these were excluded from the analysis. Organisms with less than 25 ribosomal protein annotations were discarded from further analysis. 4362 organisms remained (Table S1).

### Detection of SD and MSD sequences

SD and MSD sequences were identified using the free_scan.pl program (51), as described previously (29), except that an extended ASD (i.e. 16S nucleotides 1532–1544) was used. This program enables calculation of the free energy of pairing between the ASD and mRNA as a function of register. For SD detection, we scanned across the TIR, incrementally increasing the distance between the 5’ end of the ASD (16S nucleotide 1532) and the first nucleotide of the start codon. As a control, we applied the same method on a window 25–40 nt upstream from the start codon, too far away to contain an authentic SD. The resulting ‘hits’ are termed mock-SD (MSD) sequences. We note that in some rare instances true SD sequences are missed due to incorrect annotations of the 5’ ends of the ribosomal proteins.

### TIR affinity histograms

Histograms displaying the frequency of SD (or MSD) sequences as a function of pairing free energy for the ribosomal protein genes of the Bacteroidia were generated using MatPlotLib (52). Cumulative histograms of ASD-mRNA binding affinities of all protein-coding genes of the 200 Flavobacteriales with unique ASD were generated separately for the SD and MSD regions. The step size of these cumulative histograms was 1 kcal/mol, and they were plotted with a logarithmic y-axis using MatPlotLib. In order to estimate the maximal TIR affinity expected by chance, each cumulative histogram for the SD region was fitted with an exponential function in the range from –6 kcal/mol to –1 kcal/mol using a custom python program and the binding energy at which this exponential function reached one was denoted the expected maximal TIR affinity of the organism. All genes with a stronger TIR affinity than this expected maximal TIR affinity were classified by the difference between their TIR affinity and the expected maximal TIR affinity of their organism and for thresholds on this difference in multiples of 1 kcal/mol it was counted how many organisms have how many genes that exceed the expected maximal TIR affinity by at least the threshold.

### Annotation of TIR affinity on phylogenetic trees

Phylogenetic trees using the GTDB taxonomy system were generated using iTOL (53) to show all organism's minimum free energy behavior color coded by one of the following classifications: *rpsU* is the minimum pairing free energy and this minimum pairing free energy is stronger than –13 kcal/mol, *rpsU* is not the minimum pairing free energy or its minimum pairing free energy is weaker than –13 kcal/mol.

### Analysis of Chlorobia proteins

Sequences for bS21, bS18 and bS6 were retrieved from the NCBI database and aligned as described (29). To build a structural model of the Chlorobia 30S platform, structures of bS21, bS18 and bS6 from *Chlorobaculum tepidum* TLS were independently predicted, using a simpler version of AlphaFold v2.1.0 (54) via a Colab notebook (AlphaFold Colab). These models were then structurally aligned to the *F. johnsoniae* ribosome (PDB: 7jil) in PyMOL (v2.5.2; Schrodinger, LLC), and residues positioned to interact with 16S rRNA were inferred by inspection.

## RESULTS

### Mutations of rpsU in F. johnsoniae

**Figure 2.**
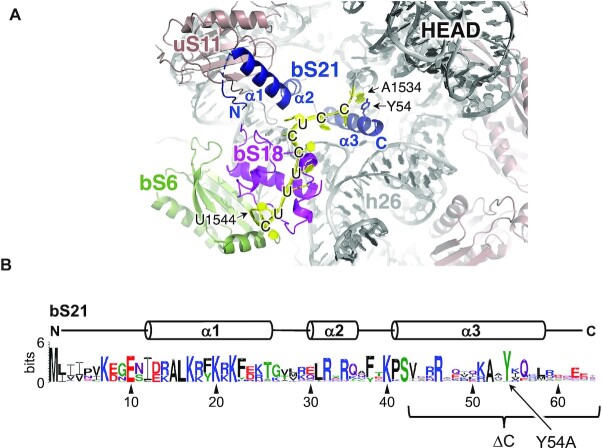
Occlusion of the ASD on the platform domain of the 30S subunit in the Bacteroidia. (**A**) A view of the *F. johnsoniae* ribosome, showing interactions of the 3’ tail of 16S rRNA (A1534-U1544; yellow, sequence indicated) with ribosomal proteins bS21 (dark blue; alpha helices and N- and C-termini labeled), bS18 (magenta), and bS6 (pea green) on the 30S platform. (**B**) Primary sequence logo for bS21 of the Bacteroidia. Residues corresponding to alpha helices (α1, α2, α3) and targeted by mutation (ΔC, Y54A) are indicated.

To investigate the role of bS21 in translational control, we first targeted the *rpsU* gene on the *F. johnsoniae* chromosome. In the *F. johnsoniae* ribosome, the C-terminal portion of bS21 primarily interacts with the 3’ tail of 16S rRNA (Figure 2). Tyrosine 54, a residue uniquely conserved in the Bacteroidia, stacks onto A1534 and appears to redirect the 3’ tail of 16S rRNA towards bS18 and bS6 on the 30S platform. Using precise allelic replacement, we deleted the DNA corresponding to codons 43–64, yielding strain ZAM64. This strain, which encodes the truncated protein bS21ΔC, is viable and forms colonies somewhat smaller than wildtype (WT) (Supplementary Figure S1). Separately, we replaced codon 54 of *rpsU* with an alanine codon. This strain, ZAM65, which encodes the mutant protein bS21(Y54A), forms colonies indistinguishable from WT.

Attempts to make the null mutant were unsuccessful, suggesting that the N-terminal portion of bS21 is essential. This prompted us to make a strain in which *rpsU* could be conditionally expressed. The native promoter on the chromosome was replaced with an engineered promoter, consisting of P*_ompA_* flanked by *lac* operators. The resulting strain, ZAM66, lacks the *lacI* gene and hence *rpsU* is constitutively transcribed. Colonies of ZAM66 were found to be smaller than WT, which may stem from lower activity of the synthetic promoter compared to the native promoter (Supplementary Figure S1).

Strains ZAM64 and ZAM66 showed marginally slower growth than WT in liquid CYE media, in line with colony sizes on solid media. Growth of ZAM65 was indistinguishable from WT, at least under the conditions tested (Supplementary Figure S1).

### Depletion of bS21 in *F. johnsoniae* causes loss of viability

To generate a strain of *F. johnsoniae* in which *rpsU* (bS21) expression can be controlled, a plasmid carrying the *E. coli lacI* gene was moved into ZAM66. Transconjugants were only obtained on plates containing the inducer, IPTG, suggesting that bS21 is needed for growth. In plating assays, cells of this depletion strain formed 10 000 times more colonies on media with IPTG than on media without IPTG (Supplementary Figure S2A). Presumably, the few colonies seen in the absence of inducer represent spontaneous suppressors. In liquid culture, growth of the depletion strain also showed clear IPTG-dependence (Supplementary Figure S2B). When log-phase cells were washed and resuspended in media lacking IPTG, growth slowed and then stopped after ∼2 doublings. Upon prolonged incubation of such cultures, a secondary phase of growth was observed. Analysis of the resulting cells showed that IPTG-dependence had been lost, indicative of suppressors (Supplementary Figure S2C). Any loss-of-function mutation in the plasmid-borne *lacI* gene would eliminate repression, which probably explains the relatively high frequency of suppressors obtained. Regardless, these collective data indicate that bS21 (or, more accurately, at least the N-terminal portion of bS21) is needed for growth of *F. johnsoniae*.

### Mutation or depletion of bS21 increases protein production from reporters with strong SD sequences *in vivo*

To investigate the effects of the *rpsU* mutations on translation, various plasmid-borne GFP reporter gene fusions were moved into UW101 (WT), ZAM64 (ΔC), ZAM65 (Y54A), and ZAM66 (Depl.) backgrounds. Each reporter construct contains the translation initiation region (TIR) from a *F. johnsoniae* gene translationally fused to *gfp*. The resulting transformants were grown to mid-log phase, and GFP levels were determined by quantifying the fluorescence of whole cells. For the bS21-depletion strains (Depl.), cell cultures were diluted into fresh media containing or lacking IPTG and grown to an OD_600_ of ∼0.5 before fluorescence measurements were taken.

**Figure 3.**
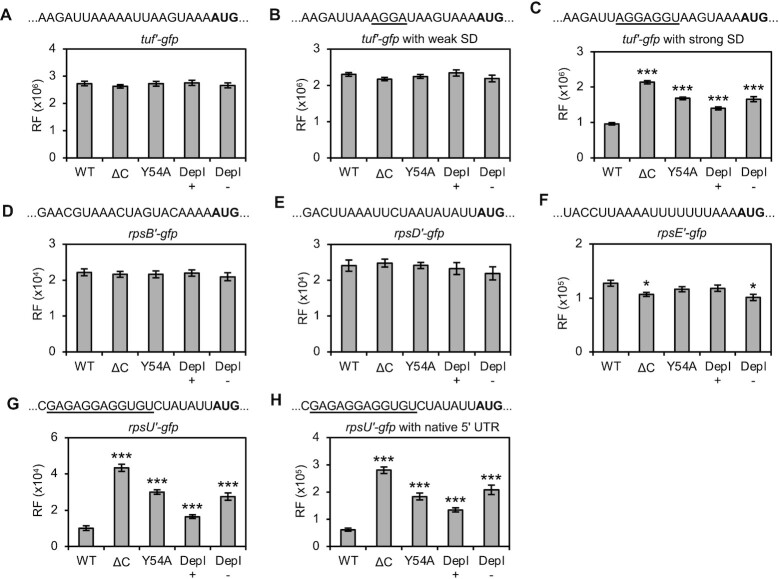
Mutation or depletion of bS21 stimulates translation of reporters with strong SD sequences *in vivo*. Production of GFP from translational fusion reporters in control (WT) and *rpsU* (ΔC, Y54A or Depl.) strains, as indicated. TIR sequences are shown at the top of each panel, with start codon in bold text and nucleotides complementary to the 3’ tail of 16S rRNA underscored. For depletion strains, cells were grown in media with (+) or without (−) added IPTG. RF, relative fluorescence. A two-tailed *t* test was used to evaluate differences from the WT. *P < 0.05; **P < 0.005; ***P < 0.0005.

The EF-Tu gene, *tuf*, naturally lacks a SD sequence and contains upstream adenines (A-3, A-6, A-12, A-13) characteristic of efficient translation in this organism (28). GFP production from *tuf’-gfp* was indistinguishable across all *rpsU* backgrounds (Figure 3A). Introduction of a weak SD sequence (AGGA) had virtually no effect, with similar levels of GFP across the board (Figure 3B). By contrast, introduction of a strong SD (AGGAGGU) changed the pattern of GFP production substantially (Figure 3C). In WT cells, levels of GFP were somewhat lower, likely due to substitutions at positions −12 and −13 and/or increased TIR secondary structure (as guanines have the highest propensity for base pairing). Relative to this benchmark, GFP production was >2-fold higher in the presence of ΔC and 70% higher in the presence of Y54A. In the depletion strain, GFP levels were 40% and 70% higher than WT in the presence and absence of IPTG, respectively (Figure 3C).

GFP synthesized from various ribosomal protein gene fusions was likewise measured in each of the strain backgrounds. The TIRs of *rpsB*, *rpsD* and *rpsE* naturally lack SD sequences, whereas *rpsU* contains an extended SD sequence. GFP production from the SD-less reporters was highly similar across all backgrounds (Figure 3D–F). For *rpsE’-gfp*, a slight decrease in GFP was seen in ΔC and depletion (-IPTG) strain cases. By contrast, GFP production from *rpsU*’*-gfp* varied by strain background (Figure 3G), just as in the *tuf* (strong SD) reporter case. GFP levels were more than 4-fold higher in the presence of ΔC, 3-fold higher in the presence of Y54A, and 3-fold higher upon depletion of bS21 (-IPTG) (Figure 3G). The depletion strain showed significantly higher GFP fluorescence even in the presence of IPTG (by 60%), in line with the *tuf* (strong SD) data. Substitution of the native promoter in this strain might reduce bS21 levels, independent of LacI, consistent with these reporter data and the growth phenotype of ZAM66. Collectively, these *in vivo* data suggest that loss of bS21 or loss of interactions between bS21 and the 3’ tail of 16S rRNA stimulates translation of mRNAs with strong SD sequences.

All reporter constructs above were made by cloning a DNA fragment encompassing the TIR region between a constitutive promoter (P_ompA_) and *gfp* in the plasmid vector, resulting in an arbitrary leader length of 178 nt (*rps* reporters) or 257 nt (*tuf* reporters). While most ribosomal genes in *F. johnsoniae* are found in operons, *rpsU* is transcribed on its own. Hence, we constructed another version of the *rpsU’-gfp* reporter such that the mRNA leader (25 nt) matched that of the native gene. With this shortened leader, there was a general increase in GFP (by ∼6-fold), but the differential pattern of GFP synthesis across the strains (Figure 3H) was virtually identical to that observed with the original construct (Figure 3G).

Note, all of these *gfp* fusions are under transcriptional control of the P_ompA_ promoter. Any effect (direct or indirect) of a given *rpsU* mutation on transcription would be expected to impact all reporters. Because no differences are seen among strains in panels A, B, D or E, it is highly unlikely that the observed differences (panels C, G and H) have a transcriptional origin.

### Ribosomes isolated from ZAM64 largely lack bS21ΔC

Tight-couple 70S ribosomes and 30S subunits were purified from wildtype and mutant strains of *F. johnsoniae*, and these particles were analyzed by SDS-PAGE and LC-MS/MS with label free quantification (LFQ) to assess protein composition (Supplementary Figure S3). In the case of ZAM64, a band corresponding to truncated bS21ΔC was observed, but considerably fainter than expected. LFQ of *N*-terminal peptides (common to all predicted bS21 variants) revealed that bS21ΔC is sub-stoichiometric, present in ∼11% of the 30S particles and ∼16% of the 70S particles. In all purified ribosomes and subunits, bS1 levels appear to be sub-stoichiometric. This is unsurprising, because bS1 is the most loosely-bound ribosomal protein, prone to dissociation during ribosome / subunit purifications (55). Compared to WT particles, bS1 seems slightly underrepresented in particles from ZAM64 and ZAM65, but a significant decrease could only be confirmed in the case of 30S subunits from ZAM65. In particles from another strain, SA04, encoding a C-terminally tagged version of bS21, levels of bS1 were more clearly down, whereas bS21 levels were unchanged (Supplementary Figure S3).

### Mutation or loss of bS21 alters the mRNA selectivity of ribosomes

**Figure 4.**
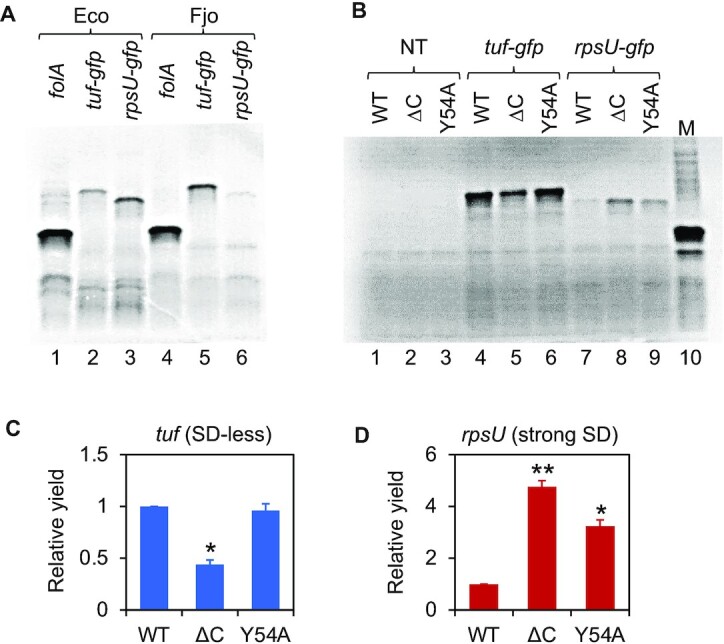
Mutation or loss of bS21 creates ribosomes with altered mRNA specificity. (**A**) The translation activity of *E. coli* ribosomes (Eco) and *F. johnsoniae* ribosomes (Fjo) was compared in the PURExpress® system. Templates included a plasmid encoding dihydrofolate reductase (*folA*) and PCR products encoding *tuf’-gfp* (SD-less) or *rpsU’-gfp* (strong SD) (as indicated; 10 ng/μL each). (**B**) The translation activity of wild-type (WT) and mutant (ΔC, Y54A) *F. johnsoniae* ribosomes in the presence of *tuf’-gfp* and *rpsU’-gfp* templates (as indicated; 33 and 50 ng/μL, respectively). NT, no template. M, size marker FolA. Relative yield of protein made by *F. johnsoniae* ribosomes (WT, ΔC, Y54A; as indicated) from *tuf’-gfp* (C) and *rpsU’-gfp* (D) templates. A two-tailed *t* test was used to evaluate differences from the WT. *P < 0.05; **P < 0.005.

To compare the translation activity of various ribosomes, we used a customized PURExpress® *in vitro* protein synthesis kit (New England Biolabs), made from purified *E. coli* components. Ribosomes and initiation factors from *F. johnsoniae* were nearly as active as those from *E. coli*, based on the yield of FolA in reactions containing the company-supplied plasmid template (Figure 4A). This shows that *F. johnsoniae* ribosomes are compatible with *E. coli* tRNAs and elongation factors. PCR product templates, containing the same *F. johnsoniae tuf’-gfp* and *rpsU’-gfp* gene fusions described above, also supported protein synthesis in this system, albeit at lower efficiency. With *E. coli* ribosomes, more translation was observed with *rpsU’-gfp* (strong SD) than *tuf’-gfp* (SD-less); whereas with *F. johnsoniae* ribosomes, the opposite trend was seen. These data are consistent with earlier evidence that *E. coli* ribosomes and *F. johnsoniae* ribosomes are intrinsically different—the latter being generally recalcitrant to SD recognition (29).

Next, we compared the translation activity of *F. johnsoniae* ribosomes isolated from control and mutant strains (Figure 4B–D). In the presence of the *tuf’-gfp* (SD-less) template, translation yields were similar for WT and Y54A ribosomes but 2-fold lower for ΔC ribosomes. By contrast, in the presence of *rpsU’-gfp* (strong SD), a completely different pattern was seen. WT ribosomes exhibited low protein production, while ribosomes from the mutant strains produced 5-fold (ΔC) and 3-fold (Y54A) more protein. These data suggest that mutation or loss of bS21 alters the TIR binding specificity of ribosomes.

### Loss of bS21 (or its C-terminal region) enables ribosomes to initiate more rapidly on rpsU mRNA

**Figure 5.**
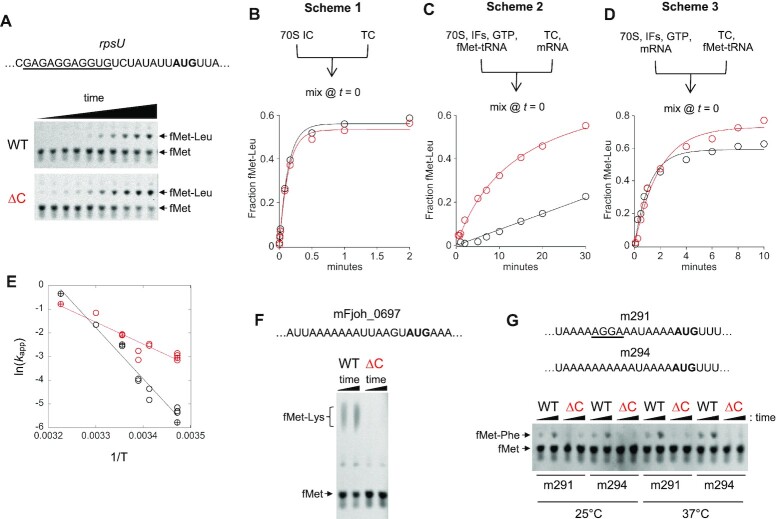
Loss of bS21 (or its C-terminus) enables ribosomes to initiate rapidly on *rpsU* mRNA. (**A**) An example of the primary data, showing formation of fMet-Leu as a function of time on wildtype (WT) and mutant (ΔC) ribosomes (as indicated). (**B–D**) Comparing the rate of dipeptide formation on WT (black circles) and ΔC (red circles) ribosomes, in the presence of *rpsU* mRNA, under different reaction schemes (as indicated), at 20°C. The apparent rate reflects decoding (scheme 1, panel B) or some step(s) of initiation (scheme 2, panel C; scheme 3, panel D). TC, EF-Tu•GTP•Leu-tRNA ternary complex; IFs, IF1 and IF2 and IF3. (**E**) Arrhenius plot comparing the temperature dependence of the rate of initiation (scheme 2) in WT (black circles) and ΔC (red circles) ribosomes in the presence of *rpsU* mRNA. Open symbols, reactions performed in TNKM buffer; crossed symbols, reactions performed in polymix buffer. T, temperature in Kelvin. (**F**) Comparison of initiation on message mFjoh_0697 (SD-less, relevant region shown) by WT and ΔC ribosomes, at 37°C, monitored via fMet-Lys formation (scheme 2). (**G**) Comparison of initiation on message m294 (SD-less, relevant region shown) and m291 (weak SD, relevant region shown) by WT and ΔC ribosomes, monitored via fMet-Phe formation (scheme 2) at 25°C and 37°C (as indicated).

To compare the rate of initiation on *rpsU* mRNA under various conditions, we prepared Leu-tRNA^Leu^ (cognate for codon 2, UUA) and employed a dipeptide formation assay (Figure 5). First, decoding was measured in WT and ΔC ribosomes. We separately preassembled the 70S IC and EF-Tu·GTP·Leu-tRNA ternary complex (TC), mixed the reactants at time *t* = 0, and quantified fMet-Leu formation as a function of time (scheme 1, Figure 5B). The apparent rate of decoding was virtually identical for WT (7.4 ± 0.5 min^−1^) and ΔC (7.3 ± 0.6 min^−1^) ribosomes. This rate is lower than one might expect, based on analogous experiments with *E. coli* ribosomes (56), but it is worth noting that unmodified (transcript) tRNA^Leu^ and heterologous (*E. coli*) EF-Tu were used here to form the TC. Next, we changed the reaction scheme to evaluate initiation. Messenger RNA was preincubated with the ternary complex, preventing any mRNA-ribosome interaction until *t* = 0 (scheme 2, Figure 5C). With this set up, apparent rates of dipeptide formation were much lower, indicating that initiation rate-limits the overall reaction in both cases. Notably, the inferred rate of initiation on *rpsU* mRNA was found to be ∼10-fold higher for ΔC ribosomes (0.085 min^−1^) than for WT ribosomes (0.008 min^−1^). When fMet-tRNA (rather than mRNA) was withheld from other components of the initiation complex until time *t* = 0 (Scheme 3, Figure 5D), the apparent rate increased by ∼6-fold in the ΔC case and by ∼100-fold in the WT case, eliminating any advantage for ΔC ribosomes. These data suggest that *rpsU* initiation involves a slow mRNA binding step and that ΔC ribosomes can engage *rpsU* mRNA considerably faster than WT ribosomes.

We noticed that the difference in initiation rate (scheme 2) seemed to depend on temperature, with a larger advantage for ΔC ribosomes at 15°C. Hence, we repeated the experiment multiple times at various temperatures and plotted ln(*k*_app_) versus 1/*T*, according to Arrhenius (Figure 5E). The WT reaction exhibited a larger temperature dependence, indicated by the steeper slope of the best-fit line, corresponding to an Arrhenius activation energy (*E*_a_) of 42.9 ± 2.7 kcal/mol. The Δ*C* reaction was less sensitive to temperature, as indicated by a considerably smaller activation energy, *E*_a_ = 18.3 ± 2.5 kcal/mol. We suspect that dissociation of the 3’ tail of 16S rRNA from the platform pocket requires more thermal energy in the presence of bS21 than in its absence. This could account for the different E_a_ values, since the 3’ tail must be liberated before it can pair with *rpsU* mRNA.

We also compared initiation (scheme 2) of WT and ΔC ribosomes on several other mRNAs (Figure 5F, G). On mFjoh_0697 (SD-less), m294 (SD-less), and m291 (weak SD), WT ribosomes clearly outperformed ΔC ribosomes. In fact, barely any dipeptide was formed by ΔC ribosomes in the presence of these mRNAs. Thus, loss of bS21 (or its C-terminus) stimulates initiation on *rpsU* mRNA, specifically.

### Initiation on rpsU is largely unaffected by bS1

Our SDS-PAGE data suggest somewhat lower levels of bS1 in ΔC ribosomes compared to WT ribosomes, raising the question of whether bS1 might be responsible for the effects observed. To test this, we purified *F. johnsoniae* bS1, incubated the protein (in 2-fold excess) with WT and ΔC ribosomes, and measured initiation on *rpsU* mRNA (Supplementary Figure S4). Protein bS1 had little impact on either reaction, marginally enhancing initiation by ΔC ribosomes and marginally inhibiting initiation by WT ribosomes (Supplementary Figure S4A). Thus, if anything, bS1 accentuated rather than diminished the effect of ΔC, providing strong evidence that loss of bS21 (and not bS1) is responsible for the regulation observed. Supplemental bS1 promoted initiation on the SD-less m294, as indicated by a 2-fold increased reaction amplitude in the WT case (Supplementary Figure S4B). Increased product was also detected in the ΔC case, although low signal precluded accurate quantification of this change. Collectively, these data suggest that bS1 contributes to initiation on certain mRNAs more than others, and initiation on *rpsU* mRNA and its regulation is largely bS1-independent.

### Wild-type and mutant ribosomes form unusually stable 70S ICs on rpsU mRNA

**Figure 6.**
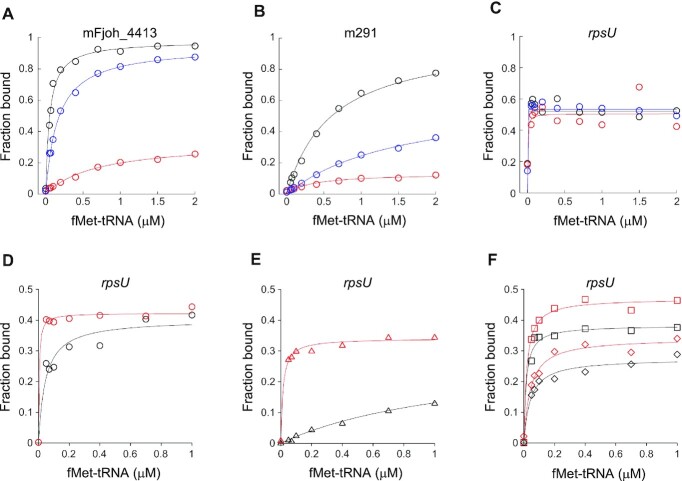
Comparison of 70S and 30S complex formation by control and mutant particles. (A–C) Toeprinting was used to measure 70S IC formation on mFjoh_4413 (**A**), m291 (**B**) and *rpsU* mRNA (**C**) by control (WT, black circles) and mutant (ΔC, red circles; Y54A, blue circles) ribosomes as a function of fMet-tRNA concentration. All reactions contained initiation factors (1.5 μM each) and GTP (100 μM). The extent of complex formation (*F*_max_) and overall equilibrium association constant (*K*_A_) were deduced by curve fitting. For panel A, WT: *F*_max_ = 0.98, *K*_A_ = 19 μM^−2^; Y54A: *F*_max_ = 0.95, *K*_A_ = 6.1 μM^−2^; ΔC: *F*_max_ = 0.34, *K*_A_ = 1.4 μM^−2^. For panel B, WT: *F*_max_ = 1.0, *K*_A_ = 1.7 μM^−2^; Y54A: *F*_max_ = 0.64, *K*_A_ = 0.64 μM^−2^; ΔC: *F*_max_ = 0.13, *K*_A_ = 3.0 μM^−2^. For panel C, all curves: *F*_max_ ≈ 0.5, *K*_A_ > 100 μM^−2^. (D, E) Toeprinting was used to measure 30S complex formation on *rpsU* mRNA for WT (black symbols) and ΔC (red symbols) subunits in the presence of all initiation factors (D), IF1 and IF2 (E), IF2 and IF3 (F, squares), or IF3 only (F, diamonds). GTP (100 μM) was present in all reactions. For panel D (all IFs), WT: *F*_max_ = 0.40, *K*_A_ = 22 μM^−2^; ΔC: *F*_max_ = 0.42, *K*_A_ > 100 μM^−2^. For panel E (IF1,IF2), WT: *F*_max_ = 0.30, *K*_A_ = 0.80 μM^−2^; ΔC: *F*_max_ = 0.34, *K*_A_ = 72 μM^−2^. For panel *F* squares (IF2,IF3), WT: *F*_max_ = 0.38, *K*_A_ = 67 μM^−2^; ΔC: *F*_max_ = 0.47, *K*_A_ = 54 μM^−2^. For panel F diamonds (IF3), WT: *F*_max_ = 0.27, *K*_A_ = 24 μM^−2^; ΔC: *F*_max_ = 0.34, *K*_A_ = 24 μM^−2^. Note, fitted curves for the most stable complexes (e.g. ΔC in panels D and E) provide only rough estimates of *K*_A_.

To further compare the ribosomes activities, we used toeprinting to evaluate 70S IC formation at 37°C as a function of fMet-tRNA concentration (Figure 6A–C). In all cases, we observed a single toeprint corresponding to the expected 70S IC, with the correct start codon in the P site (Supplementary Figure S5A). On the SD-less message mFjoh_4413, complex formation was most efficient with WT ribosomes (*F*_max_ = 0.98, *K*_A_ = 19 μM^−2^), somewhat less efficient with Y54A ribosomes (*F*_max_ = 0.95, *K*_A_ = 6.1 μM^−2^), and least efficient with ΔC ribosomes (*F_max_* = 0.34, *K*_A_ = 1.4 μM^−2^) (Figure 6A). On m291, which has a weak SD, the efficiency of 70S IC formation followed the same trend, with WT > Y54A > ΔC (Figure 6B). Interestingly, on *rpsU* mRNA, complex formation was qualitatively different than on the other mRNAs (Figure 6C). With WT, Y54A, and ΔC ribosomes, near-maximal complex formation was seen at the lowest concentration of fMet-tRNA tested (0.05 μM). Remarkably, even in the absence of fMet-tRNA, a toeprint of 10–30% of the maximal signal intensity was observed (Figure 6C, Supplementary Figure S5B). This tRNA-independent toeprint is indistinguishable from that seen in the presence of fMet-tRNA, indicating accurate positioning of the start codon in the P site. These data indicate tight binding of *rpsU* mRNA in these 70S ICs, suggesting that an extended SD-ASD helix forms in all cases under these conditions.

### Loss of bS21 (or its C-terminal region) stabilizes 30S complexes on rpsU mRNA

The experiments above showed that 70S IC formation on *rpsU* mRNA is highly favorable thermodynamically, which we reasoned could mask functional differences between the WT and mutant ribosomes. To explore this possibility, we measured formation of various 30S complexes, representing potential intermediates of initiation. On *rpsU* mRNA, ΔC subunits generally formed more stable complexes than WT subunits (Figure 6D–F). In the presence of all IFs, the *K*_A_ was clearly larger for ΔC than for WT (Figure 6D). When IF3 was omitted, an even larger difference in *K*_A_ was seen (Figure 6E). Interestingly, differences between WT and ΔC complexes were less pronounced upon omission of IF1 or omission of IF1 and IF2 (Figure 6F). Under these conditions, *K*_A_ values were comparable for WT and ΔC reactions, although ∼20% higher *F*_max_ values were seen for ΔC. These data suggest a complex interplay between factor binding, ASD sequestration, SD-ASD pairing, and fMet-tRNA binding. For WT subunits, complex formation depends on IF3 and appears to be inhibited by IF1; for ΔC subunits, complex formation is IF3-independent and stimulated by IF1. Overall, these findings suggest that loss of bS21 (or its C-terminus) stabilizes various 30S complexes on *rpsU* mRNA, corresponding to potential intermediates of initiation.

In analogous experiments, we measured 30S complex formation on mFjoh_4413, a SD-less message from *F. johnsoniae* (Supplementary Figure S6). On this mRNA, complex formation was strictly IF3-dependent and more favorable for WT subunits than ΔC subunits. In the presence of all factors, WT subunits exhibited a >2-fold higher *F*_max_ value than ΔC subunits, a difference which increased to 9-fold upon omission of IF1. These data suggest that loss of bS21 (or its C-terminus) compromises 30S and 70S IC formation on SD-less mRNAs, in line with the defects in initiation described above.

### Extended SD sequences are characteristic of rpsU

Jha *et al.* used eight nucleotides of 16S rRNA (1534–1541) to screen for SD sequences in various Bacteroidia. Strong SDs (≤–7 kcal/mol) were found frequently upstream of certain ribosomal genes, including *rpsU* (Flavobacteriales, Chitinophagales, Cytophagales, Sphingobacteriales) and/or *rpsR* (Bacteroidales, Cytophagales, Sphingobacteriales). We have since noticed that complementarity between the 3’ tail of 16S rRNA and *rpsU* extends further in many cases, up to 13 bp for some organisms (Supplementary Figure S7). This prompted us to revisit the computational screen for SDs, using virtually the entire 3’ tail of 16S rRNA (nt 1532–1544) extracted from the genome of each organism analyzed. To assess the frequency of false positives, we also screened for ‘mock SD’ (MSD) sequences, using a window too far from the start codon to contain an authentic SD. The results generally reflected those of Jha *et al.* but gave a larger overall range of potential SD pairing free energies (Supplementary Figure S8–S11). Remarkably, rRNA-mRNA pairing was found to be unusually extensive for *rpsU*, with ΔG values in the –14 to –21 kcal/mol range, corresponding to SD-ASD helices of 9 to 13 consecutive base pairs (Supplementary Figure S8). Less stable SD-ASD interactions are predicted for *rpsR*, with the range (–7 to –14 kcal/mol) and mode (–11 kcal/mol) of ΔG values shifted by 7 and 9 kcal/mol, respectively. Other ribosomal genes including *rpsB*, *rpsE*, *rpsG*, *rplE*, *rplF*, *rplI*, *rplW* and *rplX* also contain SD sequences in certain Bacteroidia (Supplementary Figure S8, S10). In these cases, the distribution of ΔG values resembles that of *rpsR*, with mode values between –8 to –12 kcal/mol, quite far from that of *rpsU* (–20 kcal/mol). Thus, extensive rRNA–mRNA pairing is a distinctive feature of *rpsU* in the Bacteroidia, implying that translational control of *rpsU* differs from that of other SD-containing genes in these organisms.

**Figure 7.**
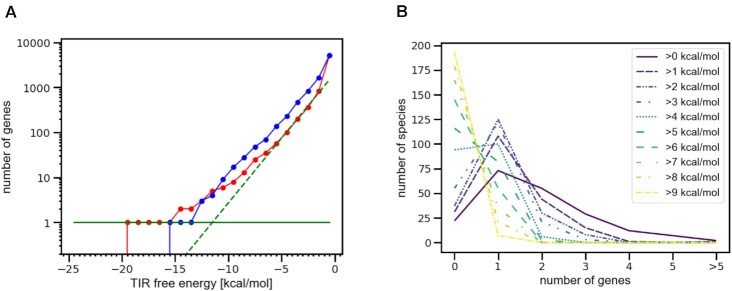
*Flavobacteriales* genomes typically have only a single gene with a strong SD. (**A**) Cumulative distributions of ASD pairing free energy for SD (red) and MSD (blue) regions in *F. johnsoniae*. The green dashed line corresponds to an exponential fit to SD-ASD pairing free energies between –6 kcal/mol and –1 kcal/mol (assumed to be coincidental and appearing as a straight line due to the logarithmic scale). The solid green line represents a gene count of one. The single gene with a TIR ASD pairing free energy of close to –20 kcal/mol and thus exceeding the point where one would expect a single gene to coincidentally have that strong a pairing free energy (the intersection of the dashed and solid green lines) by 8 kcal/mol, is *rpsU*. (**B**) Graphs showing the number of genes with excess TIR ASD pairing free energies (of variable degree, as indicated in key) for 200 *Flavobacteriales* species. Even without any further constraint on the amount of TIR ASD pairing free energy beyond the energy where one gene would be expected to have this pairing free energy by chance (>0 kcal/mol), most organisms have exactly one gene. The number of organisms with a single gene with an exceptionally strong ASD pairing free energy increases even further with excess free energy threshold and only transitions to a situation where most organisms have no gene with an exceptionally strong ASD pairing free energy beyond an excess free energy threshold of 4 kcal/mol. This indicates that the *F. johnsoniae* situation, where a single gene (*rpsU*) has an exceptionally strong SD sequence is typical in *Flavobacteriales*.

In the Flavobacteriales, SD sequences are most rare, absent from ribosomal genes except *rpsU* (29). To evaluate whether any other genes contain SD sequences, we applied free_scan to whole genomes, screening for SD and MSD ‘hits’ at various ΔG thresholds. As expected, the frequency of hits declines exponentially with increasingly stringent thresholds (Figure 7A). For *F. johnsoniae* (and virtually all Flavobacteriales species analyzed), MSD hits generally exceed SD hits, resulting in a rightward shift of the SD plot relative to the MSD plot in the exponential region. The fact that SD sequences occur at lower frequency than expected by chance suggests that selective pressure has acted to generally eliminate SD sequences in these organisms. In *F. johnsoniae*, there is one SD hit—*rpsU*—that endures well (∼8 kcal/mol) beyond the extrapolated intercept of the SD exponential curve with the *n* = 1 line, i.e. the point at which one would expect a single gene by chance (Figure 7A). An analysis across 200 Flavobacteriales shows that the situation in *F. johnsoniae* is typical in the sense that the most likely number of genes per genome with an SD hit beyond the extrapolated intercept is one and remains one even at a threshold of up to 4 kcal/mol beyond the extrapolated intercept (Figure 7B). These data suggest that SD-dependent control of translation in Flavobacteriales is limited to *rpsU* autoregulation.

### Evidence that rpsU autoregulation is widespread in the Bacteroidota

Finally, we took advantage of the unusually strong SD of *rpsU* to predict the prevalence of bS21 autoregulation across the Bacteroidota phylum. Per genome, we determined the optimal rRNA–mRNA interaction for each ribosomal protein gene and then rank-ordered the corresponding Δ*G* values. For many organisms, *rpsU* stands out as the clear ‘winner’ and exhibits pairing Δ*G* value of < –13 kcal/mol (Supplementary Figures S12 and S13). About 80% of the Flavobacteriales meet these two criteria, with most exceptions being members of the Blattabacteriaceae. Nearly all the Sphingobacteriales also meet these criteria, as do about half of the Chitinophagales. A smaller proportion (∼20%) of the Cytophagales, corresponding mainly to the Cyclobacteriaceae, meet these criteria, while no members of Bacteroidales do. Interestingly, all members of class Chlorobia meet both criteria. These observations suggest that bS21 autoregulation is widespread in the Bacteroidota, operational in class Chlorobia and in four of the five orders of class Bacteroidia.

## DISCUSSION

In this study, we report an unambiguous example of specialized ribosomes, defined as those with altered composition and function, in bacteria. In *F. johnsoniae*, the only gene with an obvious SD is *rpsU*, the gene encoding bS21. Protein bS21 contributes to the platform pocket, which binds the 3’ tail of 16S rRNA and functionally occludes the ASD. Our genetic and biochemical data provide strong evidence that ribosomes lacking bS21 enhance protein production from *rpsU* mRNA, specifically. This sets up an autoregulatory circuit, enabling bS21 to regulate its own production.

The strongest case for specialized ribosomes in eukaryotes has been presented by Karbstein and coworkers (57–59). When *Saccharomyces cerevisiae* cells are exposed to certain stresses, such as high salt or high pH, eS26 dissociates from the small subunit. These depleted subunits exhibit altered recognition of the Kozak sequence, resulting in widespread changes in protein synthesis. Among the translationally upregulated genes are those involved in stress response, enabling the cell to cope with the imposed stress. Intriguingly, although eS26 and bS21 are unrelated, the two proteins occupy the same position on the small subunit (Supplementary Figure S14). Both interact with uS11 on the platform domain and extend toward the body, forming part of the 5’ mRNA exit channel. We infer that this is a key regulatory site of the ribosome, where compositional heterogeneity can directly impact mRNA selection.

While broadly analogous, regulation by eS26 in *S. cerevisiae* differs from regulation by bS21 in *F. johnsoniae* in important ways. Loss of eS26 from ribosomes alters Kozak sequence recognition, particularly at positions –2 and –4, influencing the translation of ∼1500 genes (58). Biological outcomes of these changes include activation of the Hog1 and Rim101 pathways, which mitigate salt and pH stress, respectively. However, many genes unrelated to stress response are also impacted by loss of eS26; presumably, these are incidental consequences of the altered mRNA specificity. In the case of bS21, regulation of translation is much more precise. Only one gene—*rpsU*—contains an obvious SD in *F. johnsoniae* (and in many other Flavobacteriales), and an extended SD is necessary for stimulation of initiation by bS21-depleted ribosomes. Thus, control by these ribosomes appears to be limited to *rpsU* autoregulation. Such precise regulation relies on sequestration of the 3’ tail of the 16S rRNA in the Bacteroidia ribosome (29) and the high information content of the extended SD of *rpsU*.

Loss of bS21 (or its C-terminus) enhances the ability of *F. johnsoniae* ribosomes to translate *rpsU* mRNA, based on two different biochemical assays. Using the dipeptide assay, we show that ΔC ribosomes initiate more rapidly on *rpsU* mRNA than do WT ribosomes, an effect exacerbated at low temperature and masked at high temperature. Arrhenius analysis reveals that the activation energy for initiation on *rpsU* is substantially (24 kcal/mol) larger for WT ribosomes than for ΔC ribosomes. This is fully consistent with our model: because bS21 contributes to ASD sequestration, ribosomes lacking bS21 need less thermal energy to liberate the ASD, a prerequisite for SD-ASD pairing and efficient initiation. At 37°C, ΔC ribosomes no longer hold a rate advantage in the dipeptide assay, even though the same ribosomes maintain a clear advantage in the transcription-translation assay at this temperature. While the basis of this apparent discrepancy remains unclear, we presume that it is due to differences in reaction conditions for the two assays. Our toeprinting experiments, performed at 37°C, show that ΔC subunits form more stable pre-initiation complexes on *rpsU* mRNA than WT subunits do, a difference most pronounced in the absence of IF3. It is possible, for example, that IF3 binding partially rate-limits product formation in the transcription-translation assay but not in the dipeptide assay, which exposes or masks the effects of ΔC, respectively, at 37°C. Importantly, in *F. johnsoniae* grown at optimal temperature (30°C), protein production from *rpsU’-gfp* increases significantly when bS21 is depleted or targeted by mutation, strongly suggesting that autoregulation is normally operational in the cell.

The ΔC strain, ZAM64, grows fairly well, and ribosomes from this strain largely lack bS21ΔC. These results may seem at odds with the essentiality of bS21, but we caution against overinterpreting the data in hand. Removal of the C-terminus of bS21 certainly compromises its interaction with the ribosome, but the stoichiometry of bS21ΔC on ribosomes in the cell remains unknown. For example, bS21ΔC could be efficiently incorporated but loosely bound, prone to dissociate during ribosome / subunit purification. The fact that cell growth requires at least the *N*-terminal portion of S21 indicates that ribosomes containing bS21ΔC are functionally distinct from those completely lacking S21. Both are predicted to be deficient in ASD sequestration, but how they otherwise differ in activity remains unclear. We consider it fortuitous that ribosomes purified from the ΔC strain largely lack bS21ΔC, as bS21-deficient ribosomes correspond to the physiologically-relevant subpopulation of the autoregulatory cycle (Figure 1). Further characterization of the ΔC strain and an improved depletion strain (which grows normally in the presence of inducer) will help resolve these open questions and clarify the mechanism of bS21 autoregulation.

ΔC ribosomes exhibit enhanced activity on *rpsU* mRNA but reduced activity on all other mRNAs tested (i.e. *tuf* mRNA, mFjoh_0697, m291, m294). This implies that bS21 plays an important role in initiation on most mRNAs in *F. johnsoniae*, but the nature of this role remains ill defined. It is possible that bS21-dependent sequestration of the 3’ tail of 16S rRNA generally enhances initiation on SD-less mRNAs. A recent mutagenesis study lends some support to this idea (60). While mutations of the ASD are generally well tolerated in *F. johnsoniae*, base substitutions predicted to disrupt interactions with the platform pocket are most deleterious. Another possibility is that bS21 plays structural roles—contributing to the mRNA binding channel and/or constraining subunit dynamics—which are particularly critical in the absence of SD-ASD pairing.

Our genetic, biochemical, and bioinformatic data suggest that autoregulation of bS21 in the Bacteroidia requires an extraordinarily strong SD sequence. Why is this so? One possibility is that annealing between SD and ASD facilitates release of the 3’ tail from the platform pocket, and an extended SD-ASD helix is needed to fully dislodge the 3’ tail from the pocket. Another possibility is that initiation on *rpsU* is atypical, involving formation of a stable 30S-mRNA complex prior to fMet-tRNA recruitment, and only an extended SD-ASD helix enables formation of this key 30S-mRNA intermediate. In line with this idea, preincubation of *rpsU* mRNA with the ribosome increases the apparent rate of initiation, and ribosome complexes formed in the absence of fMet-tRNA show precise positioning of the start codon in the P site. Further experiments will be needed to test these hypotheses and elucidate the exact role of the extended SD in *rpsU* initiation.

Unusually strong SD sequences are seen upstream of *rpsU* in many members of Bacteroidia and Chlorobia, suggesting that bS21 autoregulation is widespread across the Bacteroidota phylum. As in Bacteroidia, most mRNAs are SD-less in Chlorobia (26,27). No structures of Chlorobia ribosomes have been determined, and whether the ASD is inhibited in some way remains unclear. The C-terminal portion of bS21 of Chlorobia exhibits a distinct sequence signature that does not include Y54 (Supplementary Figure S15). However, 11 other residues (of bS21, bS18 or bS6) that contact the 3’ tail 16S rRNA in the *F. johnsoniae* ribosome are conserved (or conservatively-substituted) in Chlorobia. These observations hint that, if bS21 autoregulation is indeed operational in Chlorobia, the mechanism is similar but not identical to that in Bacteroidia.

Autoregulation of bS21 synthesis should (i) ensure that the process of 30S biogenesis goes to completion and (ii) promote ribosome maintenance in the cell. Recent metagenomic studies have revealed that many aquatic bacteriophages (phages) encode a paralog of bS21 (61,62). In fact, bS21 is by far the most common ribosomal protein to be encoded by a phage. It has been proposed that, during infection, the phage-encoded bS21 replaces endogenous bS21 in ribosomes, redirecting the host translation machinery to phage mRNAs (61,62). Pelagibacter phage HTVC008M’s bS21 can be incorporated into *E. coli* ribosomes *in vivo* (62), consistent with this general model. While bS21-encoding phage are phylogenetically diverse and ecologically widespread, CRISPR-based evidence points to Bacteroidia as the main hosts of bS21-encoding myoviruses (61). We speculate that these phages might encode products that facilitate bS21 exchange on the ribosome, for example a protease that targets the host bS21. If so, the autoregulation mechanism would provide a counter defense. In cells challenged by or recovering from infection, levels of host bS21 would be quickly replenished and normal translation by replete ribosomes restored. It will be of interest to test these ideas in the future and elucidate the connections between bS21, mRNA selection, and phage biology.

Protein bS21 is essential in *F. johnsoniae*, and this appears to be the case in *E. coli* as well (63). By contrast, bS21 is nonessential in *Bacillus subtilis* and in *Listeria monocytogenes*. The null mutant of *B. subtilis* grows more slowly than the parental strain and exhibits a motility defect (64,65). In *L. monocytogenes*, loss of bS21 also slows growth and additionally confers acid tolerance (66). Notably, the bS21 gene is naturally absent from many bacteria, including most Actinobacteria, Deinococcota, Thermotogae, Fusobacteria, and some Mollicutes (9). These observations indicate that role of bS21 varies, depending on the particular organism. As part of the 5’ mRNA channel, bS21 likely functions to modulate and/or tune translation, acting more as an auxiliary factor than a core ribosomal component, in line with our current evidence that bS21 can regulate translation.

A number of other bacteria encode one to three additional copies of the bS21 gene (9). These organisms include Cyanobacteria (certain species of *Cyanothece, Cyanobacterium, Gloeobacter, Nostoc, Anabaena* and *Prochlorococcus*), Alphaproteobacteria (certain species of *Bradyrhizobium, Rhodopseudomonas, Mesorhizobium, Agrobacterium* and *Rhizobium*), Gammaproteobacteria (certain species of *Francisella* and *Burkholderia*), Spirochaetota (certain species of Borrelia), Desulfobacterota (certain species of *Geobacter*) and Firmicutes (certain species of *Clostridium*). The number of paralogous bS21 genes per organism varies considerably, even among closely related species, suggesting that acquisition and/or loss of these genes are relatively frequent evolutionary events (9). The function of these paralogs is unrelated to zinc homeostasis, because bS21 does not bind Zn^2+^. We hypothesize that these paralogs can substitute for one another in the ribosome and regulate translation. A recent study of *Francisella tularensis* lends general support to this concept (67). In *F. tularensis*, any of three bS21 paralogs can be incorporated into ribosomes but only one paralog supports growth in macrophages. It will be worthwhile to explore this and other systems to understand the extent to which bS21 proteins can shape the bacterial proteome.

## DATA AVAILABILITY

The mass spectrometry proteomics data have been deposited to the ProteomeXchange Consortium via the PRIDE (68) partner repository with the dataset identifier PXD036353. The custom scripts used during data analysis are available at doi:10.5281/zenodo.7517485.

## Supplementary Material

gkad047_Supplemental_Files
