# Supplementary material for: Ribosomes lacking bS21 gain function to regulate protein synthesis in Flavobacterium johnsoniae
Source: Nucleic Acids Res. 2023 Feb 2;51(4):1927–42. doi: 10.1093/nar/gkad047 (PMC9976891; doi:10.1093/nar/gkad047)
Supplement: gkad047_Supplemental_Files [file gkad047_supplemental_files.zip › SUPFigs.pdf]

Ribosomes lacking bS21 gain function to regulate protein synthesis in *Flavobacterium johnsoniae*

Zakkary A. McNutt[1,2], Bappaditya Roy[2,3], Bryan T. Gemler[2,4], Elan A. Shatoff[2,5], Kyung-Mee Moon[6], Leonard J. Foster[6], Ralf Bundschuh[2,4,5,7,8], and Kurt Fredrick[1,2,3] *

[1]Ohio State Biochemistry Program, The Ohio State University, Columbus, Ohio 43210, USA
[2]Center for RNA Biology, The Ohio State University, Columbus, Ohio 43210, USA
[3]Department of Microbiology, The Ohio State University, Columbus, Ohio 43210, USA
[4]Interdisciplinary Biophysics Graduate Program, The Ohio State University, Columbus, Ohio 43210, USA
[5]Department of Physics, The Ohio State University, Columbus, Ohio 43210, USA
[6]Department of Biochemistry and Molecular Biology, Michael Smith Laboratories, University of British Columbia, Vancouver, British Columbia, V3T1Z4, Canada.
[7]Department of Chemistry & Biochemistry, The Ohio State University, Columbus, Ohio 43210, USA
[8]Division of Hematology, Department of Internal Medicine, The Ohio State University, Columbus, Ohio 43210, USA

This supplement contains:

Figures S1-S12

Legend for Figure S13

Figures S14-S15

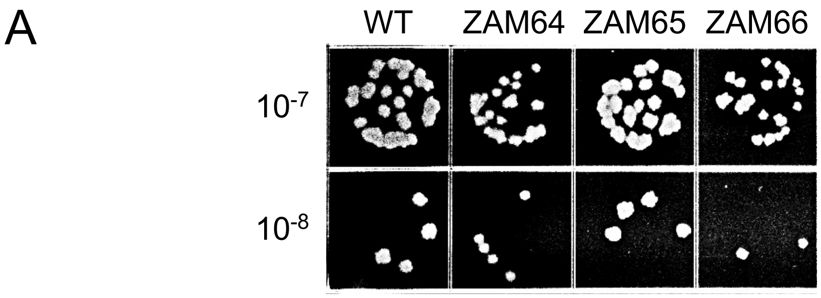

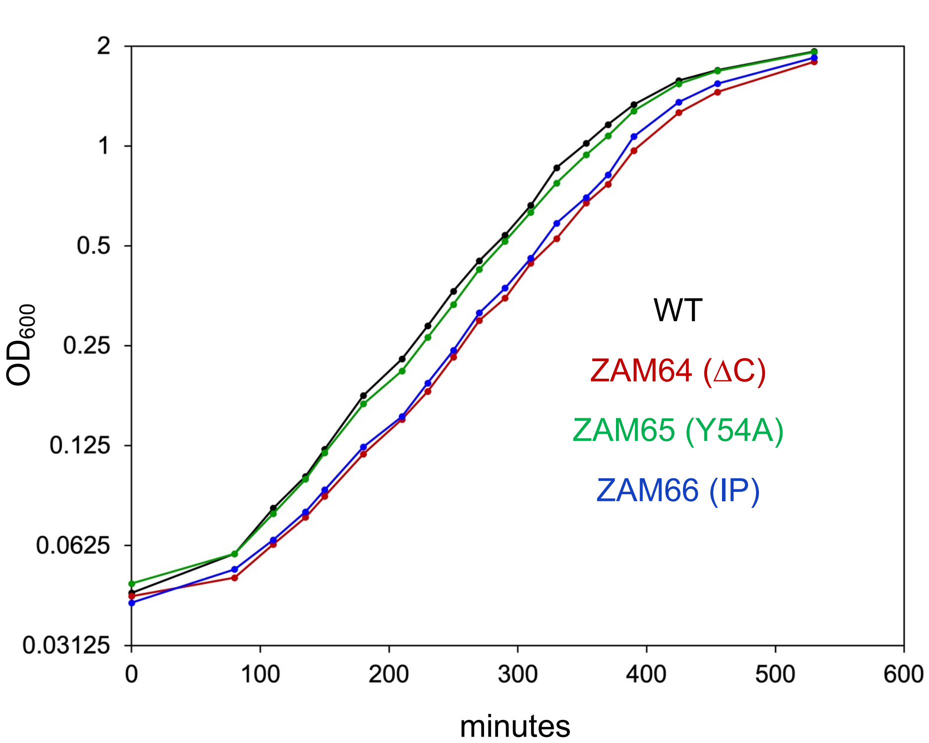

Figure S1. Effects of *rpsU* mutations on growth of *F. johnsoniae*. Control (WT) and mutant strains (ZAM64, ZAM65, and ZAM66; as indicated) were grown in parallel on solid (A) and in liquid (B) CYE media at 30°C. In panel A, cell dilutions of 10^{-7} and 10^{-8} (as indicated) were spotted, and plates were incubated for 2 days. IP, inducible promoter.

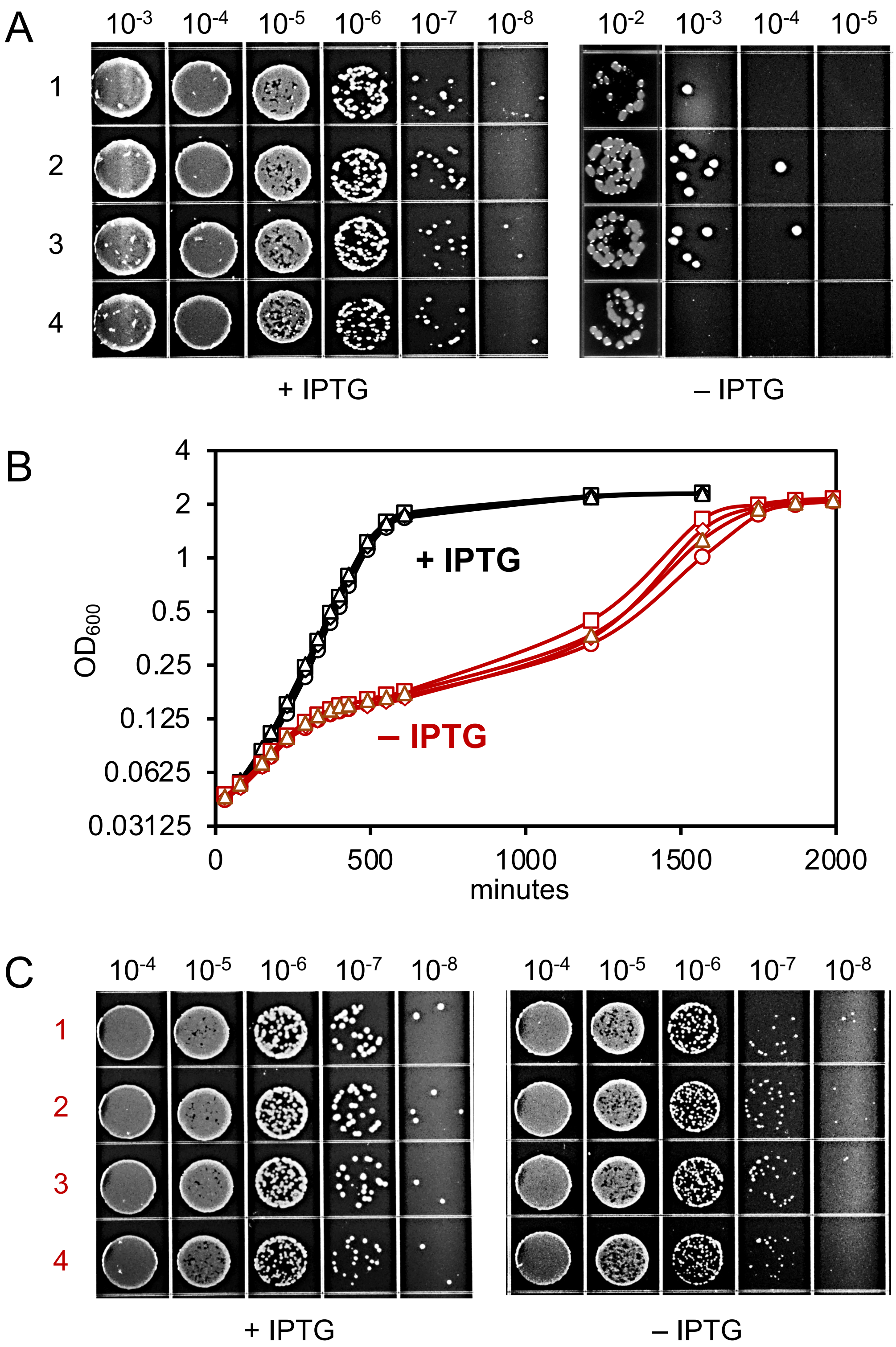

Figure S2. Effects of bS21 depletion on growth of *F. johnsoniae*. (A) Four replicated overnight cultures of the depletion strain were serially diluted (as indicated) and spotted onto solid CYE media with (+) or without (-) IPTG. Plates were incubated at 30°C for 2 days. (B) Cells of the depletion strain were used to inoculate fresh CYE media containing (black) or lacking (red) IPTG, and growth was monitored by optical density (OD_{600}). Four independent replicates (denoted by distinct symbols) were performed. (C) Cells from the saturated "-IPTG" cultures, attained after prolonged incubation (2000 min, panel B), were spotted onto solid media with and without IPTG (as indicated), and plates were incubated at 30°C for 2 days.

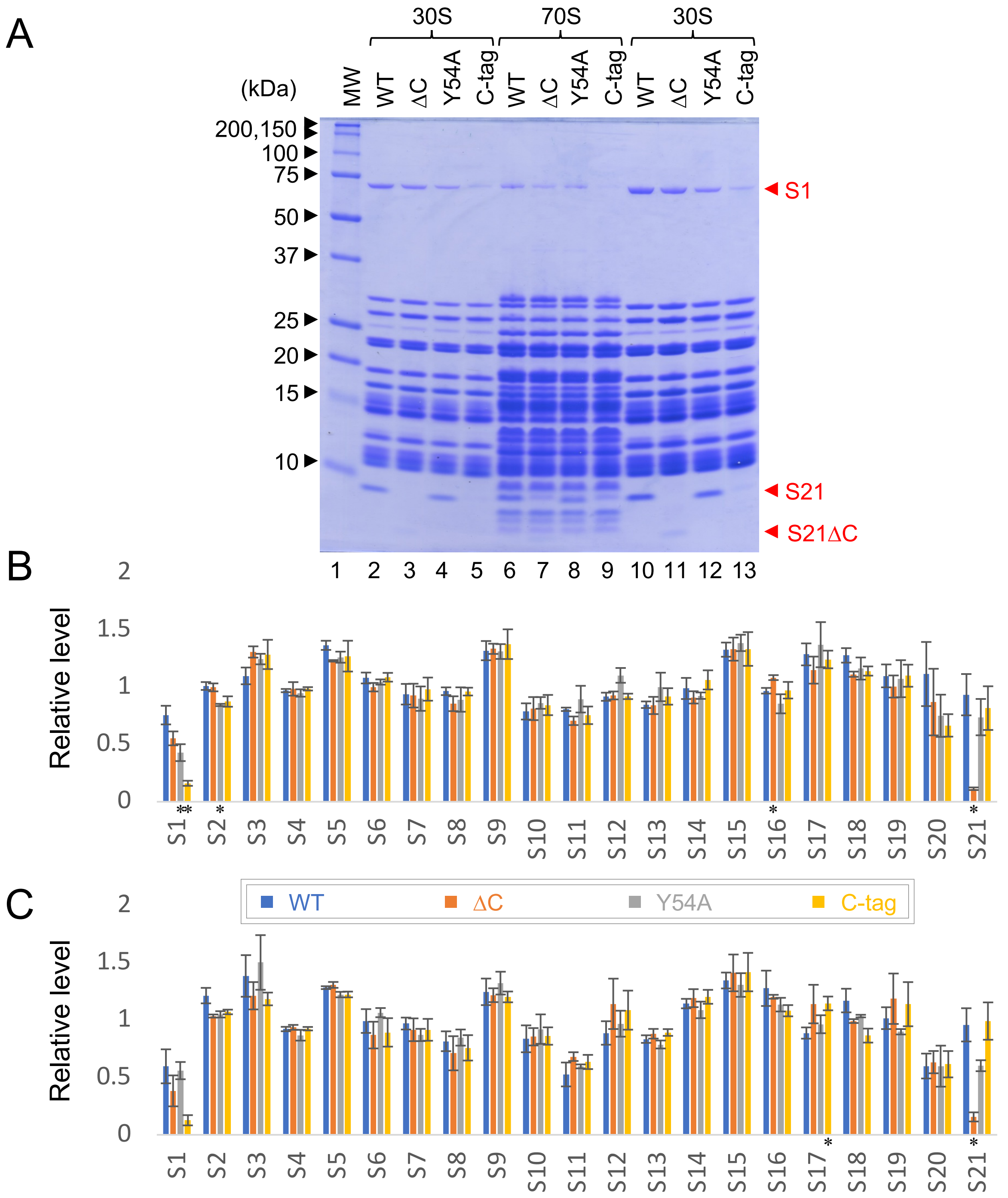

Figure S3. Protein composition of mutant ribosomes. Ribosomes and 30S subunits were purified from control (WT) and mutant strains (ΔC, Y54A, and C-tag, as indicated), and subjected to Tricine SDS-PAGE analysis (A) and LFQ LC-MS/MS (B-C). A strain encoding a C-terminally tagged version of bS21 (C-tag), used here as an additional control, will be described elsewhere. In panel A, 50 pmol of subunits or ribosomes were loaded in lanes 2-9, whereas 100 pmol of subunits were loaded in lanes 10-13. The tagged bS21 co-migrates with another protein, more slowly than the 10 kDa marker. Positions of bS1, bS21, and bS21ΔC are shown. Relative levels of small subunit proteins in 30S subunits (B) and 70S ribosomes (C) were estimated by LFQ LC-MS/MS. For S1-S20, LFQ values were normalized to the median value per sample. For S21, LFQ values for two *N*-terminal peptides (common to all protein variants) were used to determine relative levels, and data were normalized to the WT median value. Blue, WT; orange, ΔC; gray, Y54A; yellow, C-tag. Data represent the mean ± SEM for 3 technical replicates. An unpaired two-tailed *t* test was used to assess significant differences from WT. *, $p < 0.05$. For simplicity, prefixes ('u' or 'b') were omitted from protein names.

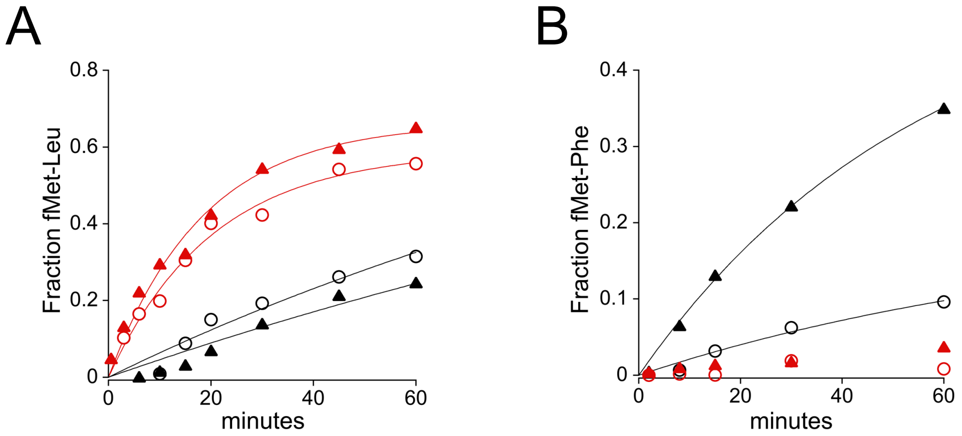

Figure S4. Effects of supplemental bS1 on initiation rates. Ribosomes (WT, black; ΔC, red) were incubated with (filled triangles) or without (open circles) 2-fold excess bS1 for 10 min at 37°C. Then apparent rates of initiation on *rpsU* mRNA at 15°C (A) or on m294 at 25°C (B) were measured via experimental scheme 2 and dipeptide formation (see Figure 5).

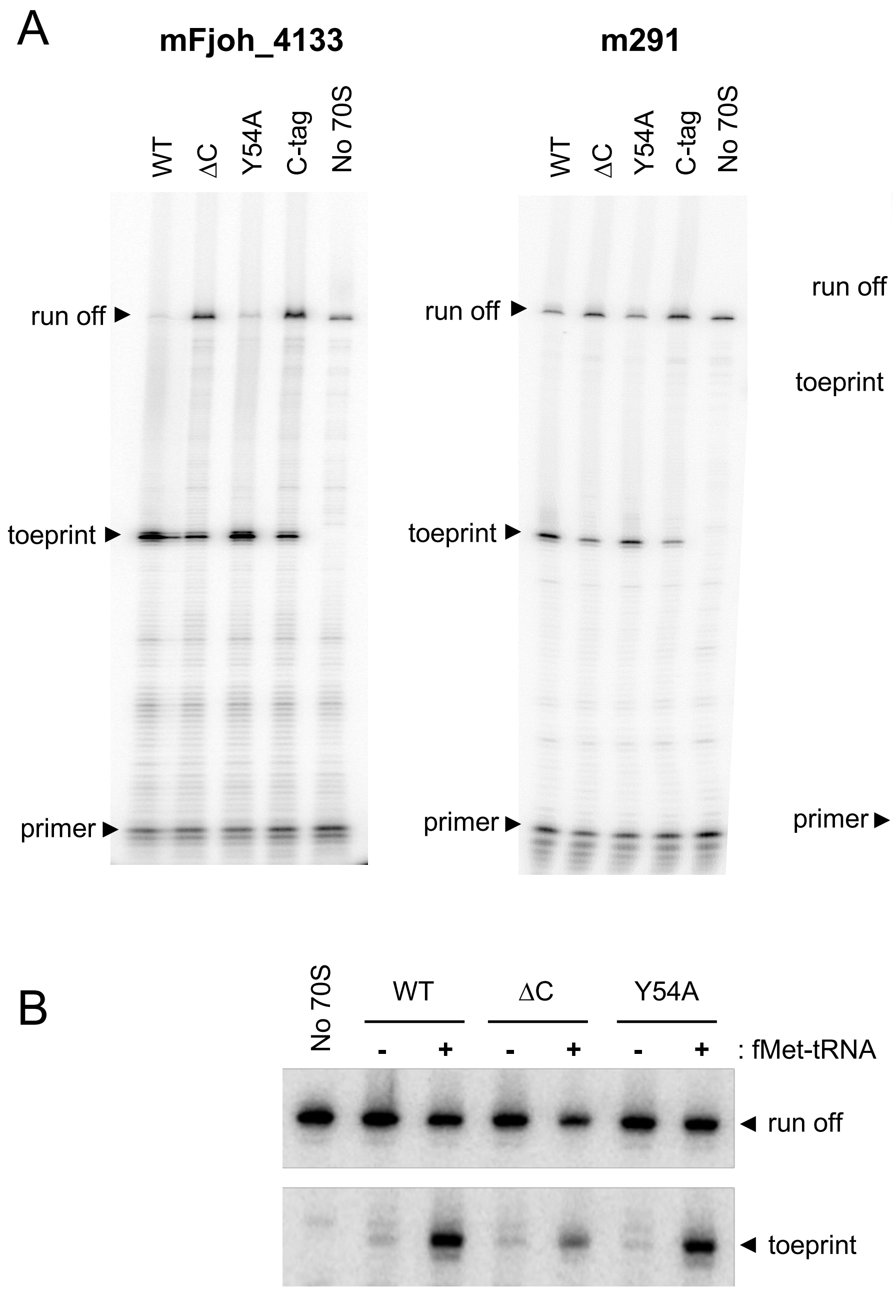

Figure S5. Detection of initiation complexes by toeprinting. (A) Examples of toeprinting gels, showing the extent of 70S IC formation by control (WT) and mutant (ΔC, Y54A, C-tag) ribosomes on three different mRNAs (as indicated). (B) Toeprinting of control (WT) and mutant (ΔC, Y54A) ribosome complexes on *rpsU* mRNA in the absence or presence of fMet-tRNA (as indicated). In these experiments, concentrations of ribosomes, fMet-tRNA, mRNA, and factors were 1 μM, 1 μM, 0.1 μM, and 2 μM, respectively.

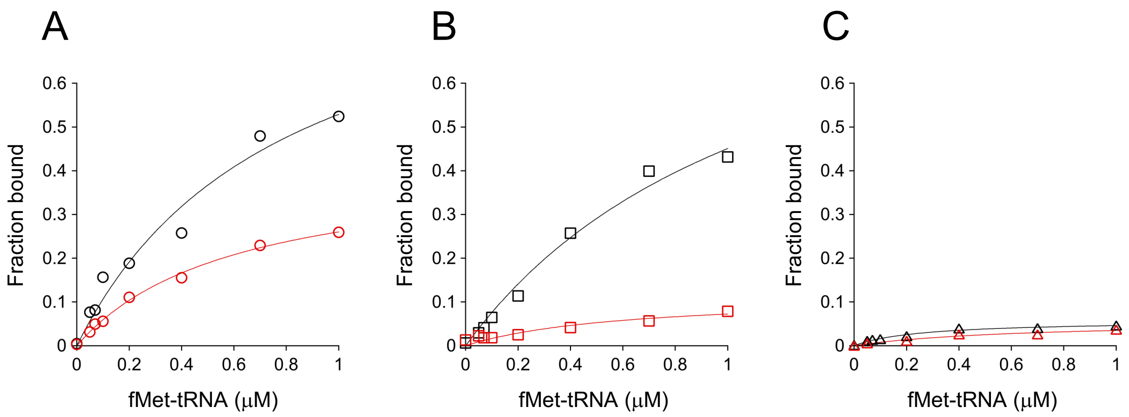

Figure S6. 30S complex formation by WT and ΔC subunits on a SD-less mRNA. Toeprinting was used to measure 30S complex formation by WT (black symbols) and ΔC (red symbols) subunits on mFjoh_4413 in the presence of all initiation factors (A), IF2 and IF3 (B), or IF1 and IF2 (C). GTP (100 μM) was present in all reactions. For panel A (all IFs), WT: F_{max} = 0.95, K_A = 1.3 μM^{-2}; ΔC: F_{max} = 0.42, K_A = 1.7 μM^{-2}. For panel B (IF2,IF3), WT: F_{max} = 1.0, K_A = 0.82 μM^{-2}; ΔC: F_{max} = 0.12, K_A = 1.7 μM^{-2}. For panel C (IF1,IF2), WT: F_{max} = 0.06, K_A = 3.9 μM^{-2}; ΔC: F_{max} = 0.06, K_A = 1.6 μM^{-2}.

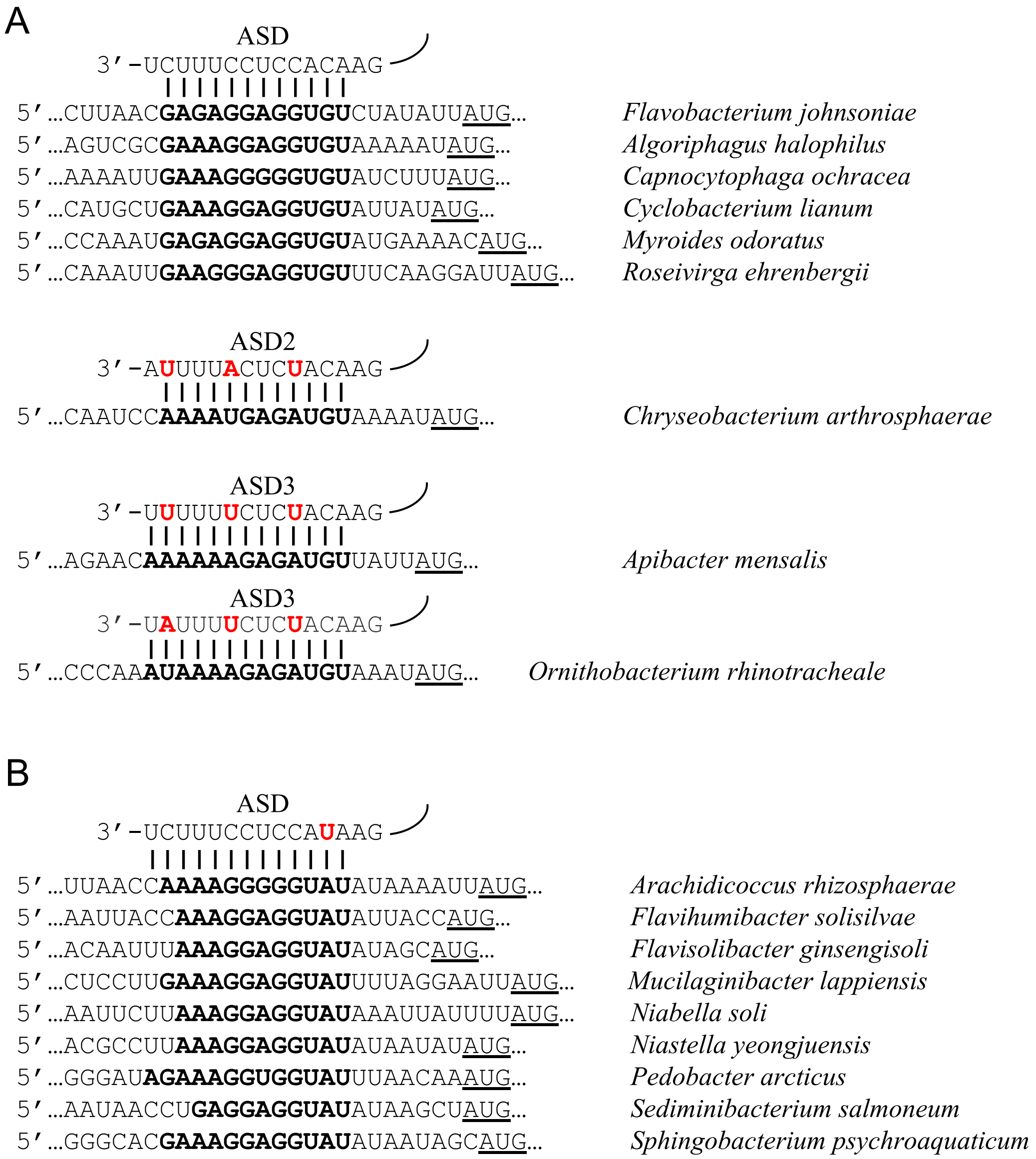

Figure S7. Extended base pairing between 16S rRNA and *rpsU* mRNA in various Bacteroidia. Complementarity between the TIR of *rpsU* and the 3' end of 16S rRNA in various organisms (as indicated). Bold text, complementarity; Underscore, start codon. (A) Organisms with C at position 1533. Certain Flavobacteriales have substitutions (red font) in the core ASD, and the alternative sequences are named "ASD2" (Jha *et al*. 2021) and "ASD3" (this work). (B) Organisms with U at position 1533 (red font).

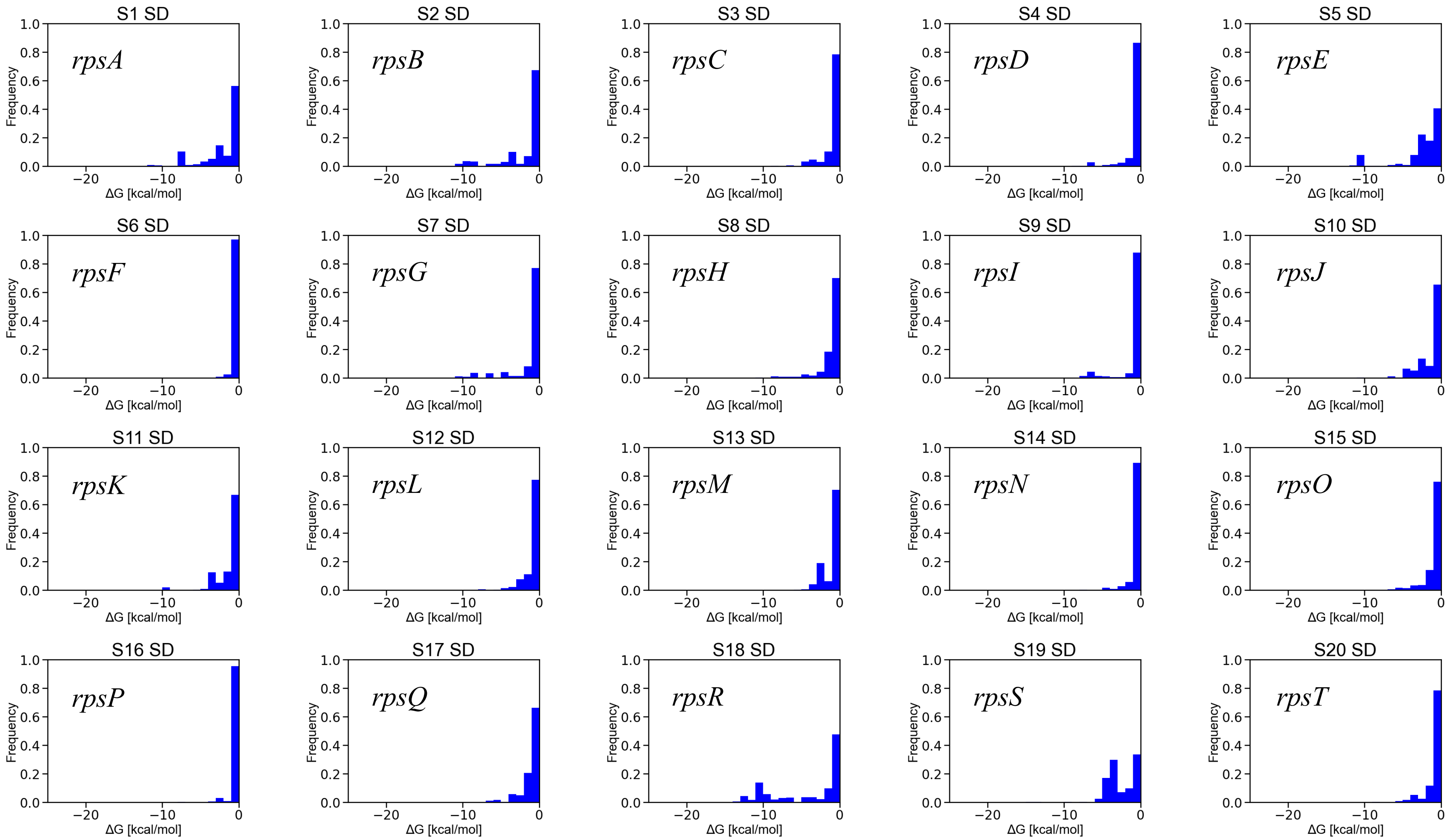

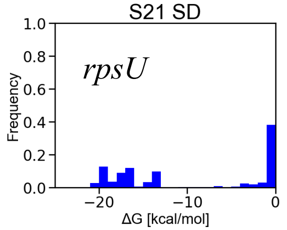

Figure S8. Frequencies of SD sequences upstream of small subunit protein genes in the Bacteroidia. Each histogram tallies the number of genes (relative frequency) for a given ribosomal protein (indicated above; gene name in panel) as a function of predicted mRNA-rRNA pairing free energy (kcal/mol). This analysis involved 378 total organisms: Flavobacteriales (n = 200), Bacteroidales (n = 49), Cytophagales (n = 73), Chitinophagales (n = 22), and Sphingobacteriales (n = 34). Contributions of each organism to the histograms depend on annotation of the respective gene, and consequently the total numbers of contributing organisms per panel (Fig. S8-S11) vary from 57 to 405.

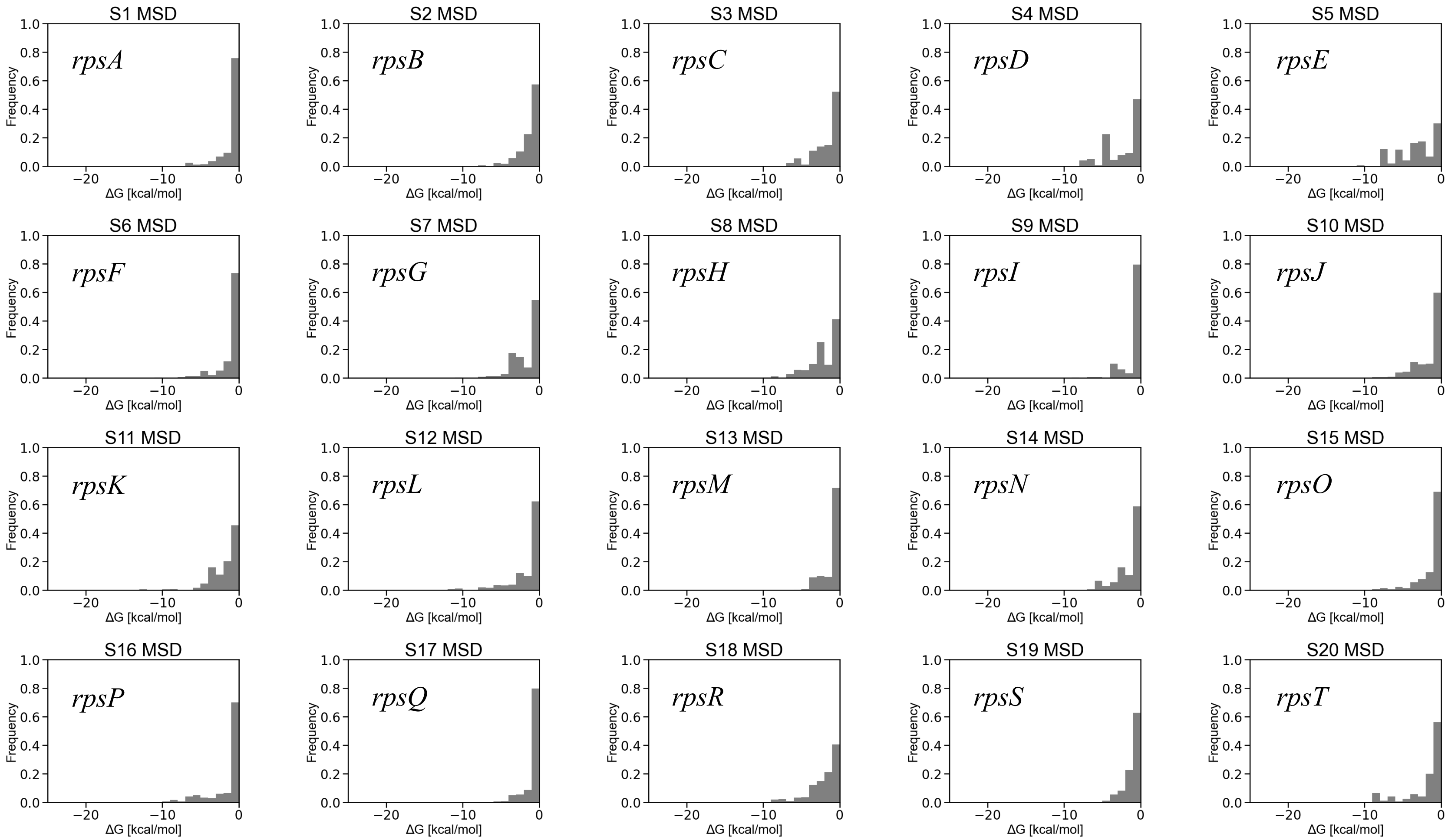

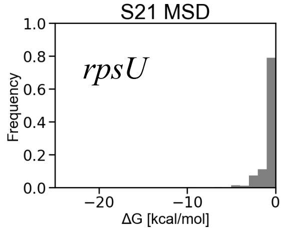

Figure S9. Frequencies of mock-SD (MSD) sequences upstream of small subunit protein genes in the Bacteroidia. Each histogram tallies the number of genes (relative frequency) for a given ribosomal protein (indicated above; gene name in panel) as a function of predicted mRNA-rRNA pairing free energy (kcal/mol). See legend of Fig. S8 for more details.

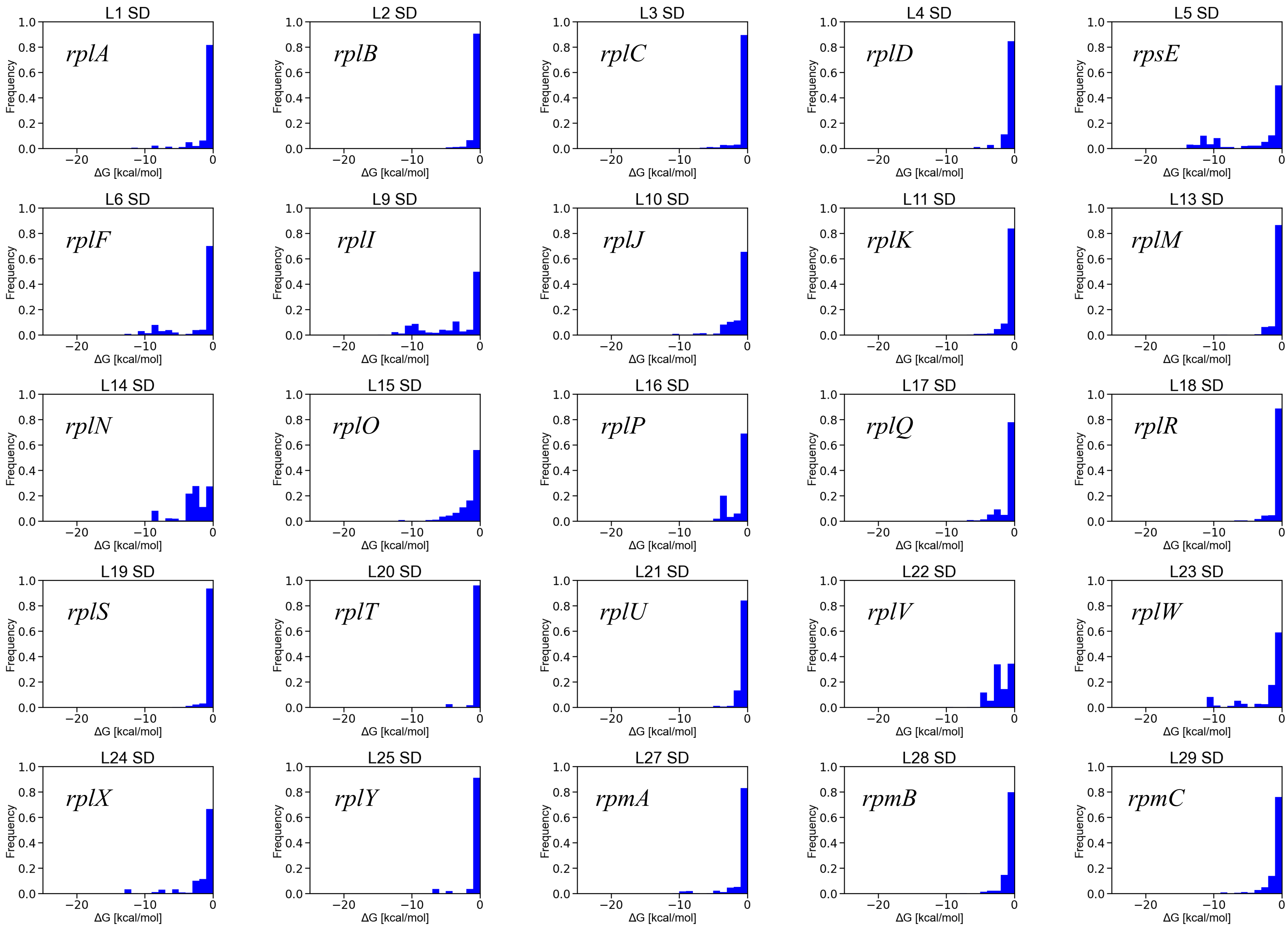

Figure S10. Frequencies of SD sequences upstream of large subunit protein genes in the Bacteroidia (continued next page).

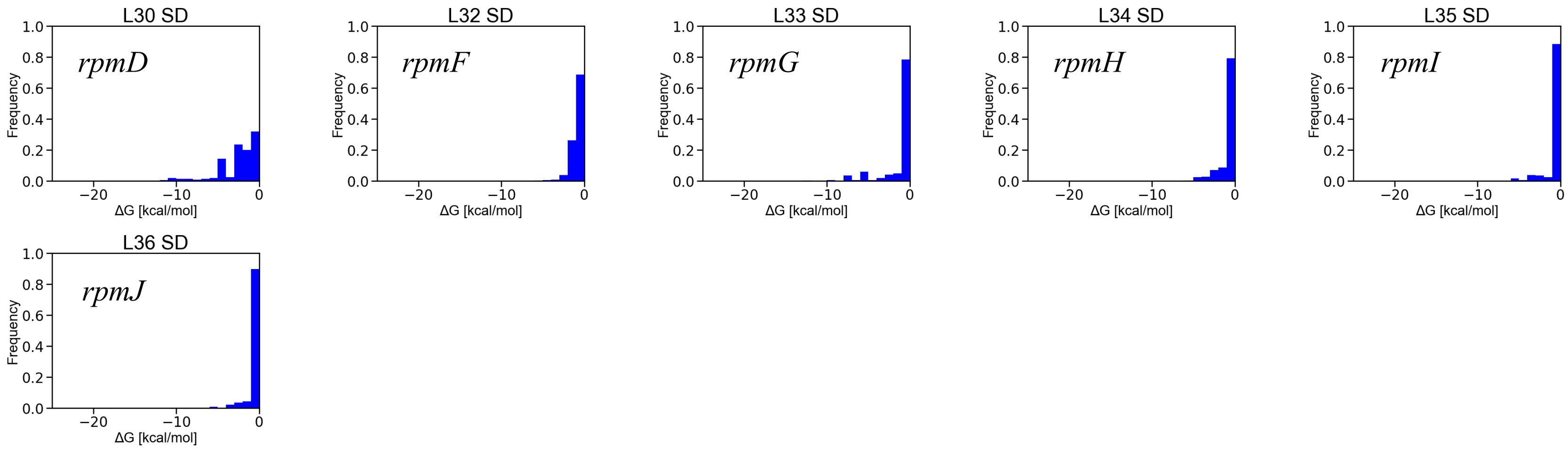

Figure S10. Frequencies of SD sequences upstream of large subunit protein genes in the Bacteroidia. Each histogram tallies the number of genes (relative frequency) for a given ribosomal protein (indicated above; gene name in panel) as a function of predicted mRNA-rRNA pairing free energy (kcal/mol). As no SD sequences were found for the L7/12 gene previously (Jha *et al.* 2021), we left it out here for technical reasons (variable annotation). See legend of Fig. S8 for more details.

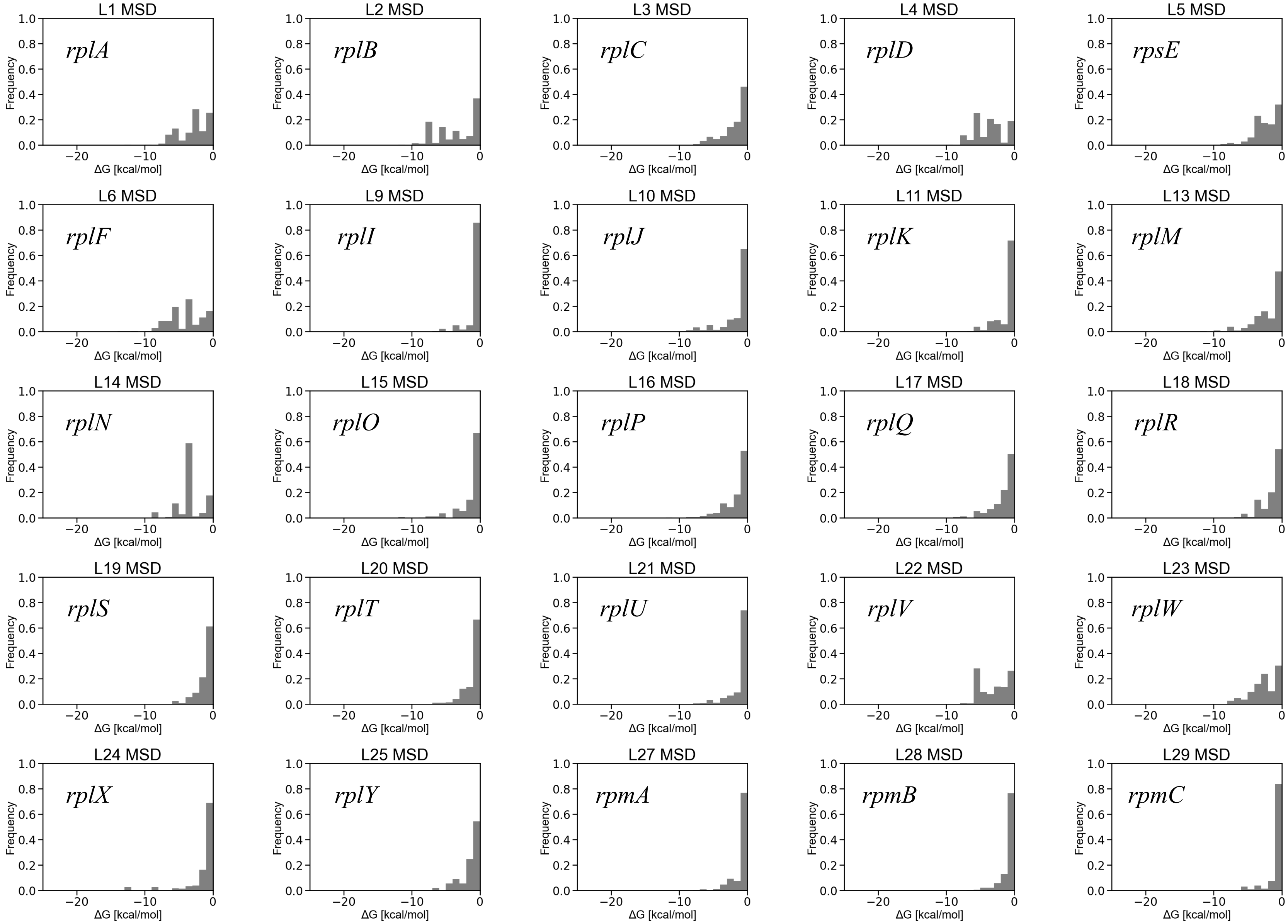

Figure S11. Frequencies of mock-SD (MSD) sequences upstream of large subunit protein genes in the Bacteroidia (continued next page).

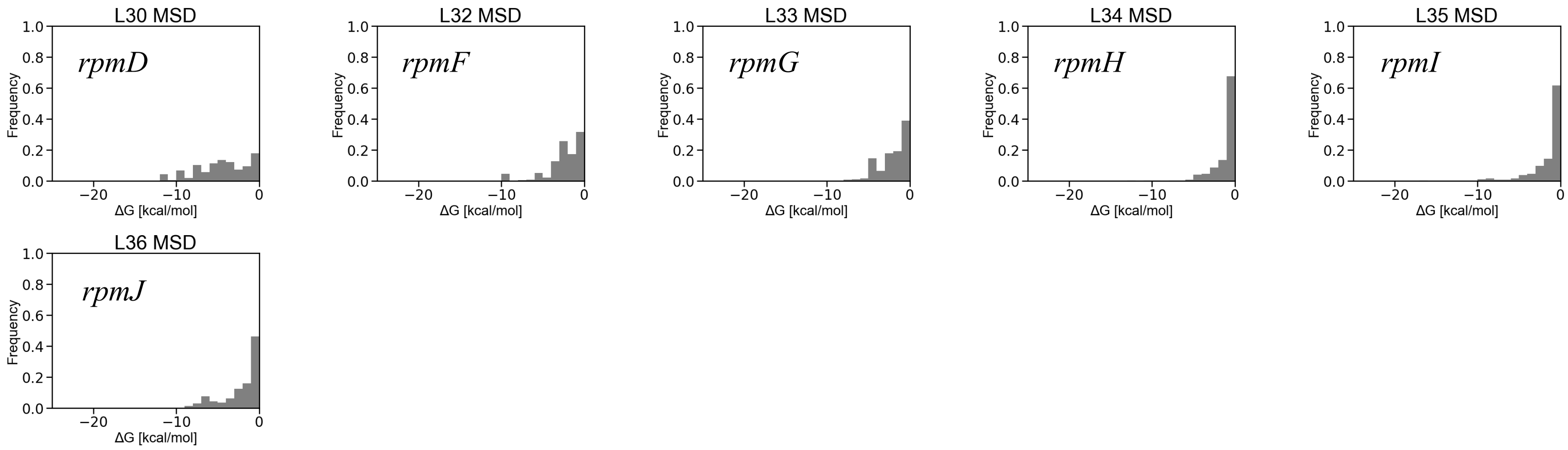

Figure S11. Frequencies of mock-SD (MSD) sequences upstream of large subunit protein genes in the Bacteroidia. Each histogram tallies the number of genes (relative frequency) for a given ribosomal protein (indicated above; gene name in panel) as a function of predicted mRNA-rRNA pairing free energy (kcal/mol). See legend of Fig. S8 for more details.

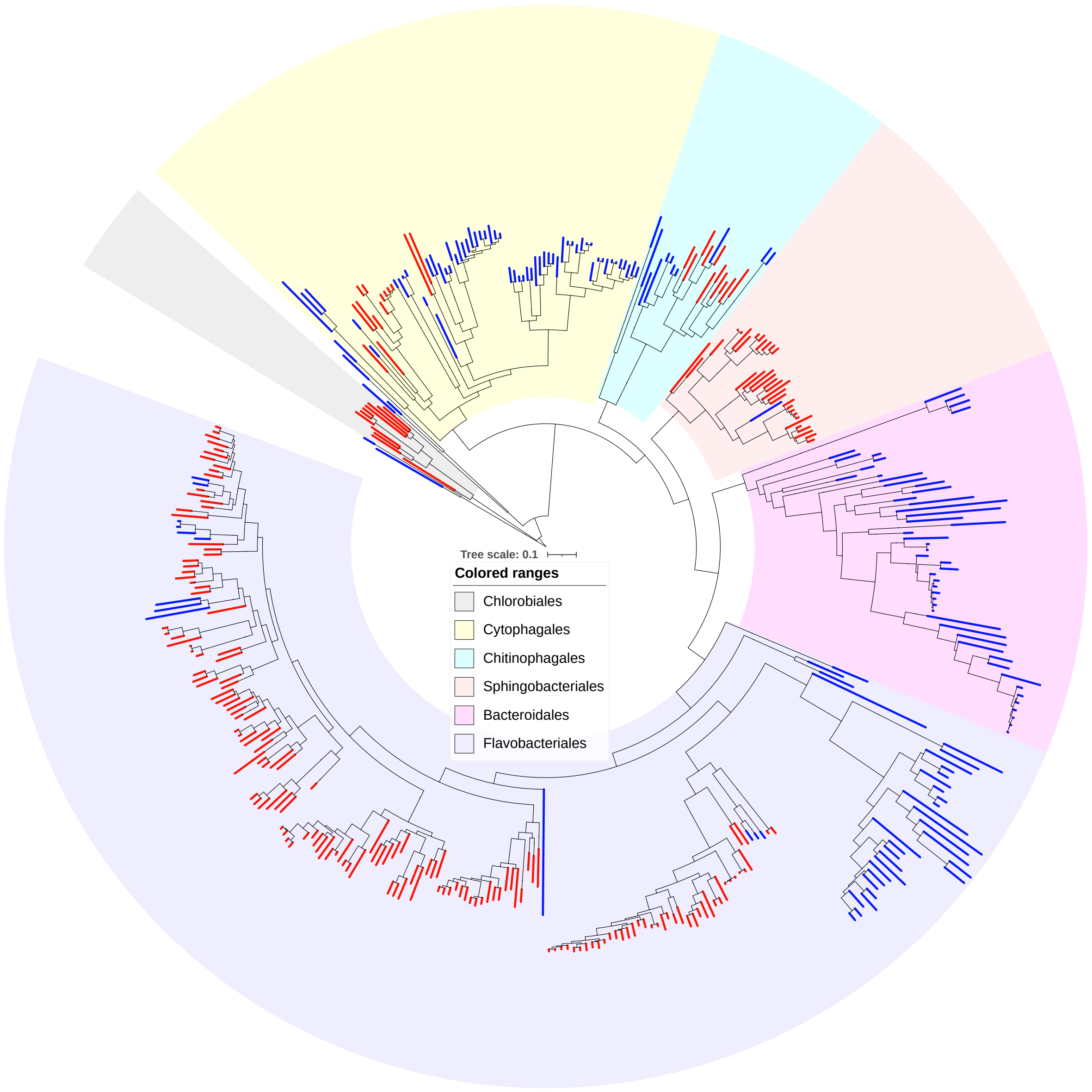

Figure S12. Predicted distribution of *rpsU* autoregulation across the Bacteroidota. Phylogenetic tree showing the predicted prevalence and distribution of *rpsU* autoregulation. Red leaves: organisms in which *rpsU* has the "strongest" SD among the ribosomal protein genes, and the *rpsU* SD-ASD helix exhibits a ΔG of < -13 kcal/mol. Blue leaves: organisms that fail to meet either of these criteria. A more detailed version of this tree listing species names and ΔG values is part of the online supplement (Fig. S13).

Legend for Figure S13 (see file: Figure_S13_tree.pdf)

Figure S13. Predicted distribution of *rpsU* autoregulation across the Bacteroidota. A more detailed version of the phylogenetic tree of Fig. S12, showing the predicted prevalence and distribution of *rpsU* autoregulation. Red leaves: organisms in which *rpsU* has the “strongest” SD among the ribosomal protein genes, and the *rpsU* SD-ASD helix exhibits a ΔG of < -13 kcal/mol. Blue leaves: organisms that fail to meet either of these criteria. Names of species and ΔG values for the *rpsU* SD-ASD interaction are listed.

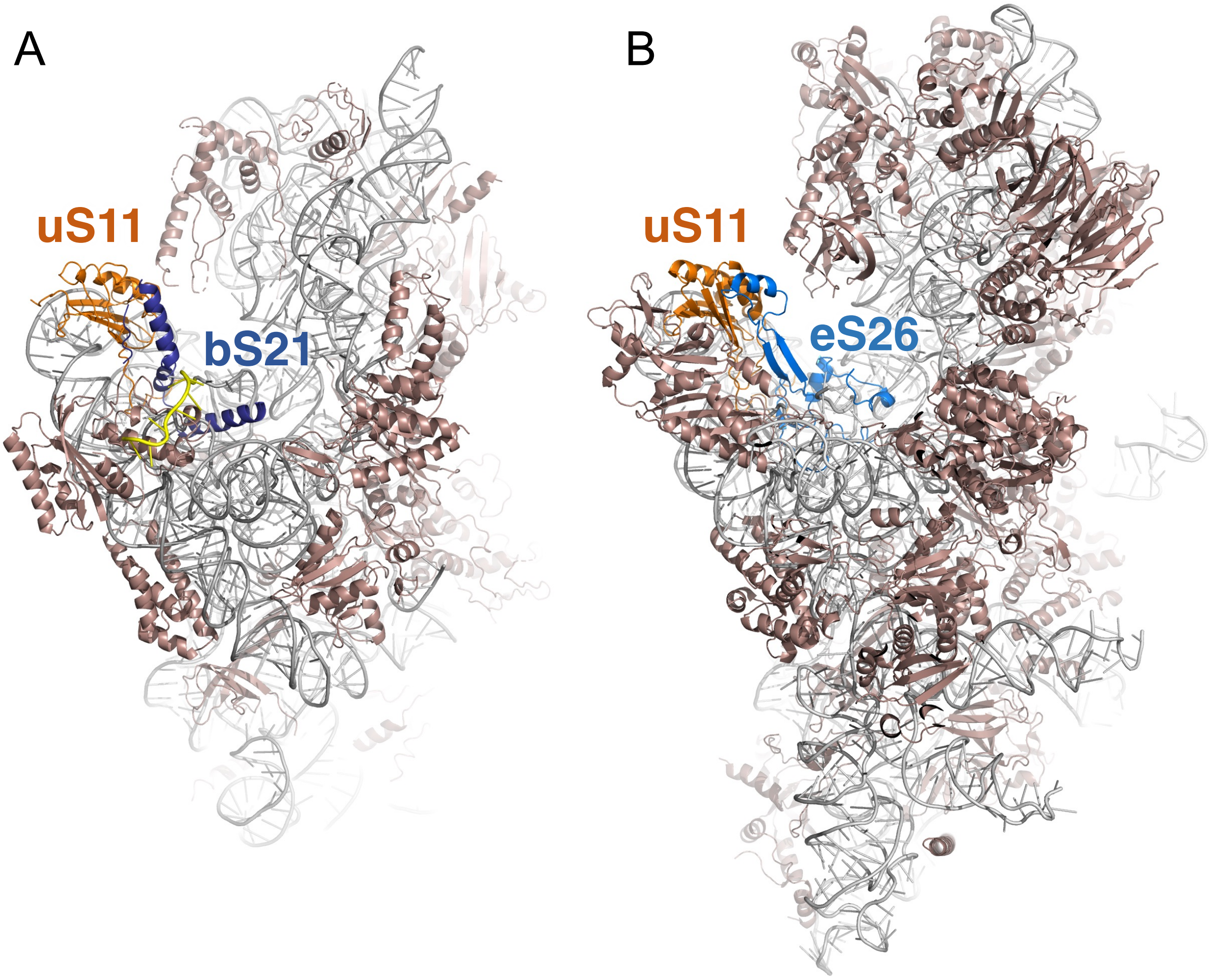

Figure S14. Proteins bS21 and eS26 occupy the same position on the small subunit. Structures of the small subunit of the *F. johnsoniae* (A) and *S. cerevisiae* (B), viewed from the E-site side. Proteins uS11 (orange), bS21 (dark blue), and eS26 (royal blue) are indicated, and nucleotides 1534-1544 of 16S rRNA are highlighted yellow. Images based on PDB files 7JIL and 7A1G.

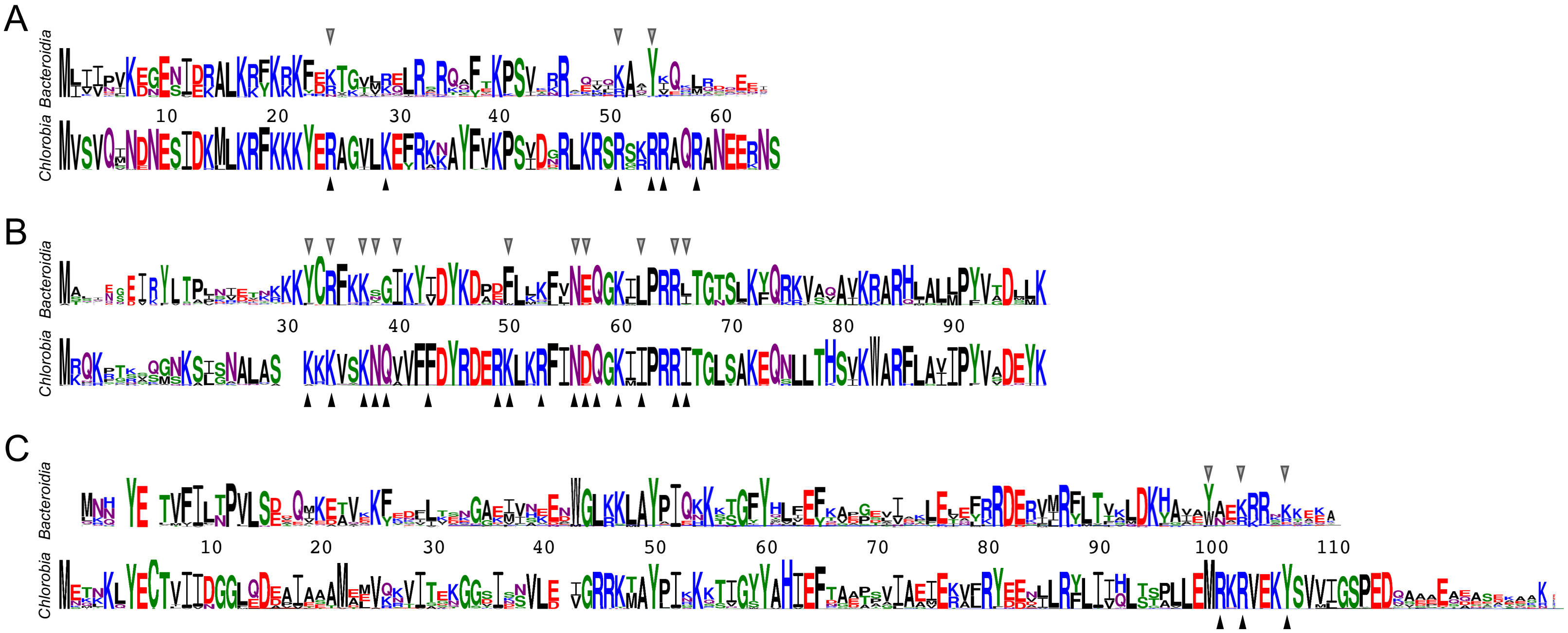

Fig. S15. Evidence that the 30S platform of the Chlorobia ribosome contains the tail-binding pocket. Aligned sequence logos of bS21 (A), bS18 (B), and bS6 (C) from Bacteroidia and Chlorobia. Bacteroidia, *n* > 300; Chlorobia, *n* > 40. Gray arrowheads show the residues that contact the 3' tail of 16S rRNA in the *F. johnsoniae* structure (PDB: 7JIL). To evaluate the Chlorobia platform, a structural model was generated using AlphaFold and PyMOL (see Materials and Methods). Black arrowheads show residues of *Chlorobaculum tepidum* proteins positioned to interact with the 3' tail of 16S rRNA.
