# Supplementary figures and images for: Ribosomes lacking bS21 gain function to regulate protein synthesis in Flavobacterium johnsoniae
Source: Nucleic Acids Res. 2023 Feb 2;51(4):1927–42. doi: 10.1093/nar/gkad047 (PMC9976891; doi:10.1093/nar/gkad047)

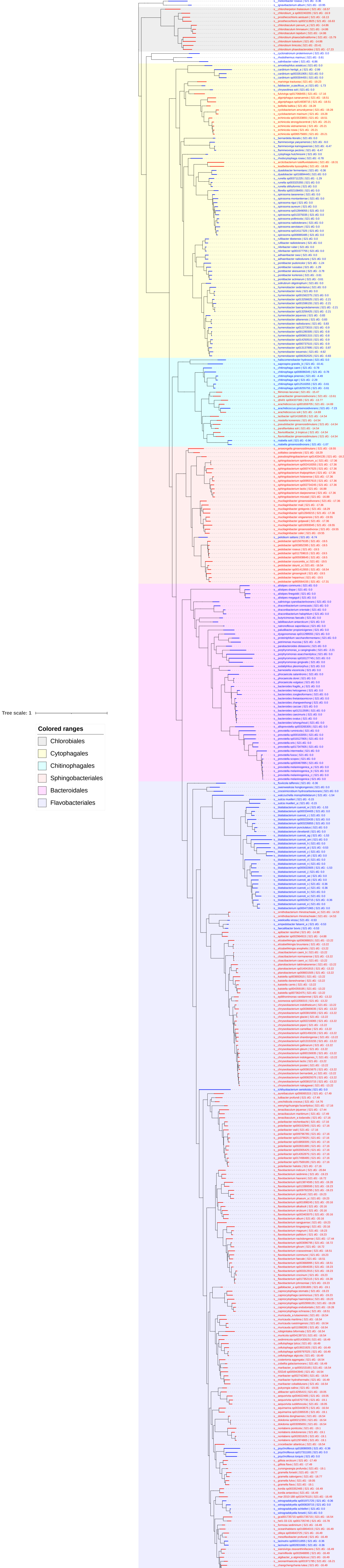

Supplement: gkad047_Supplemental_Files [file gkad047_supplemental_files.zip › Figure_S13_tree.pdf]
